# Supplementary material for: A topological refactoring design strategy yields highly stable granulopoietic proteins
Source: Nat Commun. 2022 May 26;13:2948. doi: 10.1038/s41467-022-30157-2 (PMC9135769; doi:10.1038/s41467-022-30157-2)
Supplement: Supplementary file 1 — Supplementary Information [file 41467_2022_30157_MOESM1_ESM.pdf]

Supplementary Information for

A topological refactoring design strategy yields highly stable granulopoietic proteins

Julia Skokowa[1,†,‡], Birte Hernandez Alvarez[2,†], Murray Coles[2], Malte Ritter[1], Masoud Nasri[1], Jérémy Haaf[1], Narges Aghaallaei[1], Yun Xu[1], Perihan Mir[1], Ann-Christin Krahl[1], Katherine W. Rogers[3,4], Kateryna Maksymenko[2,3], Baubak Bajoghli[1], Karl Welte[1], Andrei N. Lupas[2], Patrick Müller[3,5], Mohammad ElGamacy[1,2,3,5,‡]

[1]University Hospital Tübingen, Division of Translational Oncology, 72076 Tübingen (Germany)
[2]Max Planck Institute for Biology, 72076 Tübingen (Germany)
[3]Friedrich Miescher Laboratory of the Max Planck Society, 72076 Tübingen (Germany)
[4]Current address: Division of Developmental Biology, Eunice Kennedy Shriver National Institute of Child Health and Human Development, National Institutes of Health, Bethesda, MD, 20892 (USA)
[5]Current address: University of Konstanz, Department of Biology, 78464 Konstanz (Germany)
[6]Heliopolis Biotechnology Ltd., Cambridge, CB24 9RX (UK)

[†]Equally contributing authors
[‡]Corresponding authors

Contents

Supplementary Tables 1-5

Supplementary Movies 1-4

Supplementary Figures 1-32

Supplementary Methods

Supplementary Table 1. Proliferation potential of the tested designs obtained from the dose-response curves (Fig. 2A and Fig. 6A).

Sequence	Global sequence identity with G-CSF***	NFS-60 EC_{50}* (ng/ml)	MW (Da)	NFS-60 EC_{50} (nM)	Hill slope
>boskar1<br>LAALAAELAEIYKGLAEYQARLQSLEGISPEL<br>GPALDALRLDVADFATTLAQAMEEKKTNLPQ<br>SFLLKALEQIRKIQADAAALREKLAATYTGTD<br>RAAAAVEIAAQLEAFLEKAYEILRHLAAA	31%	2173 ± 263	13211	164.5 ± 19.9	1.05 ± 0.1
>boskar2<br>LAALAAELAEIMKGLQEYQARLKSLEGISPEL<br>GPALDALRLDMADFATTMAQMMEENPSDLP<br>QSFLLKALEQIRKIQADAAALREKLAATYPNS<br>QRAAAAVEIAAQLEAFLEKAYQILRHLAAA	31%	3225 ± 991	13339	241.8 ± 74.3	0.73 ± 0.09
>boskar3<br>LAALAAVLAEIYKGLAEYQARLQSLEGISPEL<br>GPALDALRLDVADFATTIAQAMEENKGPLPQ<br>SFLLKALEQIRKIQADAAALREKLAATYPSSQ<br>RAAAAVEIAAQLEAFLEKAYEILRHLAAA	32%	768 ± 79	13131	58.5 ± 6.0	1.12 ± 0.11
>boskar4<br>LAALAAALAEIYKGLAEYQARLKSLEGISPEL<br>GPALDALRLDMADFATTMAQAMEEGLDSLP<br>QSFLLKALEQIRKIQADAAALREKLAATYKG<br>NDRAAAAVEIAAQLEAFLEKAYQILRHLAAA	30%	27 ± 2.7	13143	2.05 ± 0.2	0.99 ± 0.09
>boskar4_t2<br>AALAAALAEIYKGLAEYQARLKSLEGISP<br>ELGPALDALRLDMADFATTMAQAMEEG<br>LDSLPQSFLLKALEQIRKIQADAAALREK<br>LAATYKGNDRAAAAVEIAAQLEAFLEKA<br>YQILRHLAAAGGGGSSGGGGSSGGGGSS<br>GGGGSSAALAAALAEIYKGLAEYQARLK<br>SLEGISPELGPALDALRLDMADFATTMA<br>QAMEEGLDSLPQSFLLKALEQIRKIQADA<br>AALREKLAATYKGNDRAAAAVEIAAQLE<br>AFLEKAYQILRHLAAA	30%	5.07 ± 0.28	27878	0.18 ± 0.01	1.03 ± 0.053
>boskar4_st2<br>AALAAALAEIYKGLAEYQARLKSLEGISPELG<br>PALDALRLDMADFATTMAQAMEEGLDSLPQ<br>SFLLKALEQIRKIQADAAALREKLAATYKGN<br>DRAAAAVEIAAQLEAFLEKAYQILRHLAAAG<br>GGGSSAALAAALAEIYKGLAEYQARLKSLEGI<br>SPELGPALDALRLDMADFATTMAQAMEEGL<br>DSLPQSFLLKALEQIRKIQADAAALREKLAAT<br>YKGNDRAAAAVEIAAQLEAFLEKAYQILRHL<br>AAA	30%	0.202 ± 0.021	26671	$7.6\times10^{-3} \pm 7\times10^{-4}$	1.722 ± 0.24

*>wild type G-CSF***<br>MSSLPQSFLLKCLEQVRKIQGDGAALQEKLC<br>ATYKLCHPEELVLLGHSLGIPWAPLSSCPSQA<br>LQLAGCLSQLHSGLFLYQGLLQALEGISPELG<br>PTLDTLQLDVADFATTIWQQMEELGMAPALQ<br>PTQGAMPAFASAFQRRAGGVLVASHLQSFLE<br>VSYRVLRHLAQP	100%	0.055	18266	0.003	

*Values obtained from 48 h treatment

** The cloned sequence represents the structured part of chain A in the PDB: 2D9Q crystal structure, starting from residue number 7. Disulfide bridged cystine are underlined.

*** BLAST alignments are shown in Table S5

Supplementary Table 2. CoMAND ensemble structure statistics

**R-Factors**[1]	
R_{mean}	0.31 ± 0.11
Coverage[2]	107/115
**Restraints**[3]	
**Distances**	
Inter-helix	73
H-bond	83
**Dihedrals**	241
**Covalent Geometry**	
Bonds (Å × 10^{-3})	1.36 ± 0.02
Angles (°)	1.43 ± 0.06
Impropers (°)	2.36 ± 0.33
**Structure Quality**[4]	
Ramachandran Map (%)	99.2 / 0.8 / 0.0
Sidechain Regularity (%)	99.0 ± 0.9
Clash Score[3]03.9 ± 1.798.1**Structure Ensemble**	
Number of Structures	17
Ordered Residues	2-53, 60-118
Backbone Heavy Atom (Å) [5]	0.80 ± 0.13
All Heavy Atom (Å) [5]	1.29 ± 0.15

[1]Ensemble R-factors averaged across the sequence (R_{mean} ± SD).

[2]The coverage refers to the number of residues used in final optimization, versus the total number expected from the sequence, excluding purification tags.

[3]Restraints used to calculate the intermediate ensemble. Note that the final ensemble is unrestrained, and individual structures may violate these restraints.

[4]Determined by MOLPROBITY [82]. The Ramachandran statistic lists the percentage of residues in favored / allowed / disfavored regions of the map (percentiles 98.0 / 99.8 / >99.8). Sidechain regularity lists the percentage in allowed sidechain rotamers (percentile 98.0). The clash score lists steric overlaps > 0.4 Å per 1000 atoms.

[5]The RMSD to the average structure based on superimposition over ordered residues, as defined in the table.

Supplementary Table 3. High-confidence interhelical (long-range) NOE contacts used in refinement.

Residue	Atom	Residue	Atom
5	HN	51	CE
5	HN	51	CG
7	HN	114	CD1
9	HN	47	CE
9	HD1	44	HA
9	HD1	48	HN
16	HN	36	CD2
15	HE1	100	HG2+
15	HE1	104	HN
15	HE1	104	HA
15	HD1	104	HD1+
15	HE1	104	HD1+
15	HE1	103	HB2
15	HE1	103	HN
15	HE2	107	HD2
15	HE2	107	HE2
19	HN	40	CE
39	HN	73	CG1
40	HN	73	CG2
40	HN	73	CD1
40	HE+	15	HB+
40	HE+	104	HD2+
43	HD1	12	HD1+
43	HD2	73	HD1+
43	HE1	8	HG2+
43	HE2	73	HD1+
43	HE2	115	HD1+
43	HZ	66	HB+
43	HZ	115	HD2+
43	HZ	115	HD1+
44	HN	12	CD2
44	HN	12	CB
46	HG2+	62	HE2
47	HA	62	HE1
47	HE+	5	HD2+
48	HN	9	CB
50	HN	62	CE2
50	HB+	62	HZ

**51**	**HN**	**5**	**CD1**
**62**	**HZ**	**47**	**HE+**
**66**	**HB+**	**43**	**HE1**
**67**	**HD2+**	**112**	**HD2**
**67**	**HD2+**	**112**	**HE2**
**67**	**HD1+**	**112**	**HD2**
**67**	**HD1+**	**112**	**HE2**
**67**	**HD1+**	**116**	**HN**
**70**	**HD1+**	**43**	**HE2**
**73**	**HG2+**	**40**	**HG1**
**73**	**HG2+**	**40**	**HG2**
**83**	**HN**	**32**	**HB+**
**100**	**HG2+**	**15**	**HD1**
**100**	**HG2+**	**15**	**HE1**
**104**	**HD1+**	**15**	**HE1**
**104**	**HD1+**	**15**	**HD1**
**104**	**HD1+**	**74**	**HA**
**107**	**HB1**	**15**	**HE2**
**107**	**HB2**	**15**	**HE2**
**107**	**HE1**	**8**	**HA**
**107**	**HE2**	**8**	**HG2+**
**107**	**HE1**	**11**	**HA1**
**107**	**HE1**	**11**	**HA2**
**107**	**HZ**	**8**	**HA**
**107**	**HZ**	**8**	**HG2+**
**107**	**HZ**	**11**	**HA1**
**107**	**HZ**	**11**	**HA2**
**107**	**HZ**	**12**	**HN**
**108**	**HD2+**	**71**	**HA**
**111**	**HB+**	**43**	**HZ**
**112**	**HN**	**70**	**HD1+**
**112**	**HD1**	**70**	**HG2+**
**112**	**HE2**	**116**	**HD+**
**115**	**HN**	**67**	**HD1***
**115**	**HD2+**	**43**	**HZ**

Supplementary Table 4. Inter-cohort neutrophil count differences in one- and 3-day-treated *Tg(mpx:GFP)* zebrafish embryos (Fig. 8A,B). p-values are shown for single-sided t-tests.

Two-tailed test	Treatment duration (day)	p-value	significant
Boskar4_t2 *vs*. Moevan_control	1	0.0146	yes
Moevan_control vs uninjected	1	0.7776	no
rhG-CSF *vs*. Moevan_control	1	<0.0001	yes
Boskar4_st2 *vs*. Moevan_control	1	<0.0001	yes
Boskar4_t2 *vs*. Moevan_control	3	0.0250	yes
Moevan_control *vs*. uninjected	3	0.0508	no
rhG-CSF *vs*. Moevan_control	3	<0.0001	yes
Boskar4_st2 *vs*. Moevan_control	3	<0.0001	yes

Supplementary Table 5. BLAST alignments of Boskar designs and rhG-CSF

Design	Alignment
Boskar1	Query 73 LHSGLFLYQGLLQALEGISPELGPTLDTLQLDVADFATTIWQQMEELG-------MAPAL 125<br>++ GL YQ LQ+LEGISPELGP LD L+LDVADFATT+ Q MEE + AL<br>Sbjct 11 IYKGLAEYQARLQSLEGISPELGPALDALRLDVADFATTLAQAMEEKKTNLPQSFLLKAL 70<br><br>Query 126 QPTQGAMPAFASAFQRRAGGVLVAS-----------HLQSFLEVSYRVLRHLA 167<br>+ + + A A+A + + + L++FLE +Y +LRHLA<br>Sbjct 71 EQIRK-IQADAAALREKLAATYTGTDRAAAAVEIAAQLEAFLEKAYEILRHLA 122
Boskar2	Query 73 LHSGLFLYQGLLQALEGISPELGPTLDTLQLDVADFATTIWQQMEE------------L 119<br>+ GL YQ L++LEGISPELGP LD L+LD+ADFATT+ Q MEE L<br>Sbjct 11 IMKGLQEYQARLKSLEGISPELGPALDALRLDMADFATTMAQMMEENPSDLPQSFLLKAL 70<br><br>Query 120 GMAPALQPTQGAM-PAFASAF---QRRAGGVLVASHLQSFLEVSYRVLRHLA 167<br>+Q A+ A+ + QR A V +A+ L++FLE +Y++LRHLA<br>Sbjct 71 EQIRKIQADAAALREKLAATYPNSQRAAAAVEIAAQLEAFLEKAYQILRHLA 122
Boskar3	Query 72 QLHSGLFLYQGLLQALEGISPELGPTLDTLQLDVADFATTIWQQMEE------------ 118<br>+++ GL YQ LQ+LEGISPELGP LD L+LDVADFATTI Q MEE<br>Sbjct 10 EIYKGLAEYQARLQSLEGISPELGPALDALRLDVADFATTIAQAMEENKGPLPQSFLLKA 69<br><br>Query 119 LGMAPALQPTQGAM-PAFASAF---QRRAGGVLVASHLQSFLEVSYRVLRHLA 167<br>L +Q A+ A+ + QR A V +A+ L++FLE +Y +LRHLA<br>Sbjct 70 LEQIRKIQADAAALREKLAATYPSSQRAAAAVEIAAQLEAFLEKAYEILRHLA 122
Boskar4	Query 72 QLHSGLFLYQGLLQALEGISPELGPTLDTLQLDVADFATTIWQQMEE------------ 118<br>+++ GL YQ L++LEGISPELGP LD L+LD+ADFATT+ Q MEE<br>Sbjct 10 EIYKGLAEYQARLKSLEGISPELGPALDALRLDMADFATTMAQAMEEGLDSLPQSFLLKA 69<br><br>Query 119 LGMAPALQPTQGAM-PAFASAFQ---RRAGGVLVASHLQSFLEVSYRVLRHLA 167<br>L +Q A+ A+ ++ R A V +A+ L++FLE +Y++LRHLA<br>Sbjct 70 LEQIRKIQADAAALREKLAATYKGNDRAAAAVEIAAQLEAFLEKAYQILRHLA 122

Supplementary Movie 1. Representative time-lapse analysis of NFS-60 cells cultured without treatment (PBS only) for 136 h.

Supplementary Movie 2. Representative time-lapse analysis of NFS-60 cells cultured with rhG-CSF (10 ng/mL) treatment for 136 h.

Supplementary Movie 3. Representative time-lapse analysis of NFS-60 cells cultured with Boskar3 (4 μg/mL) treatment for 136 h.

Supplementary Movie 4. Representative time-lapse analysis of NFS-60 cells cultured with Boskar4 (4 μg/mL) treatment for 136 h.

Supplementary Figure 1

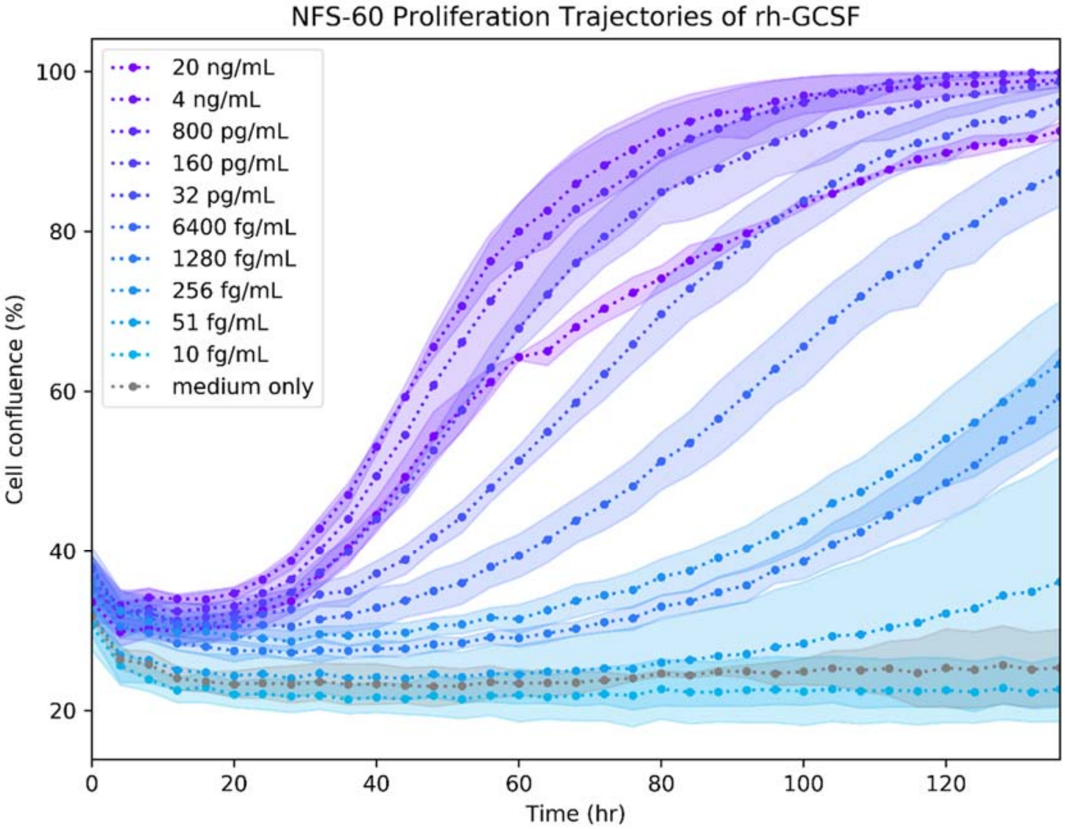

Supplementary Figure 1. Time- and dose-response curves of NFS-60 cells proliferation with rhG-CSF treatment. Dose- and time-dependent proliferation trajectories over a 5-day treatment of free-floating CD34$^+$ cells, under the influence of rhG-CSF treatment. Data points and shades represent the mean and standard deviation from 3 independent measurements.

Supplementary Figure 2

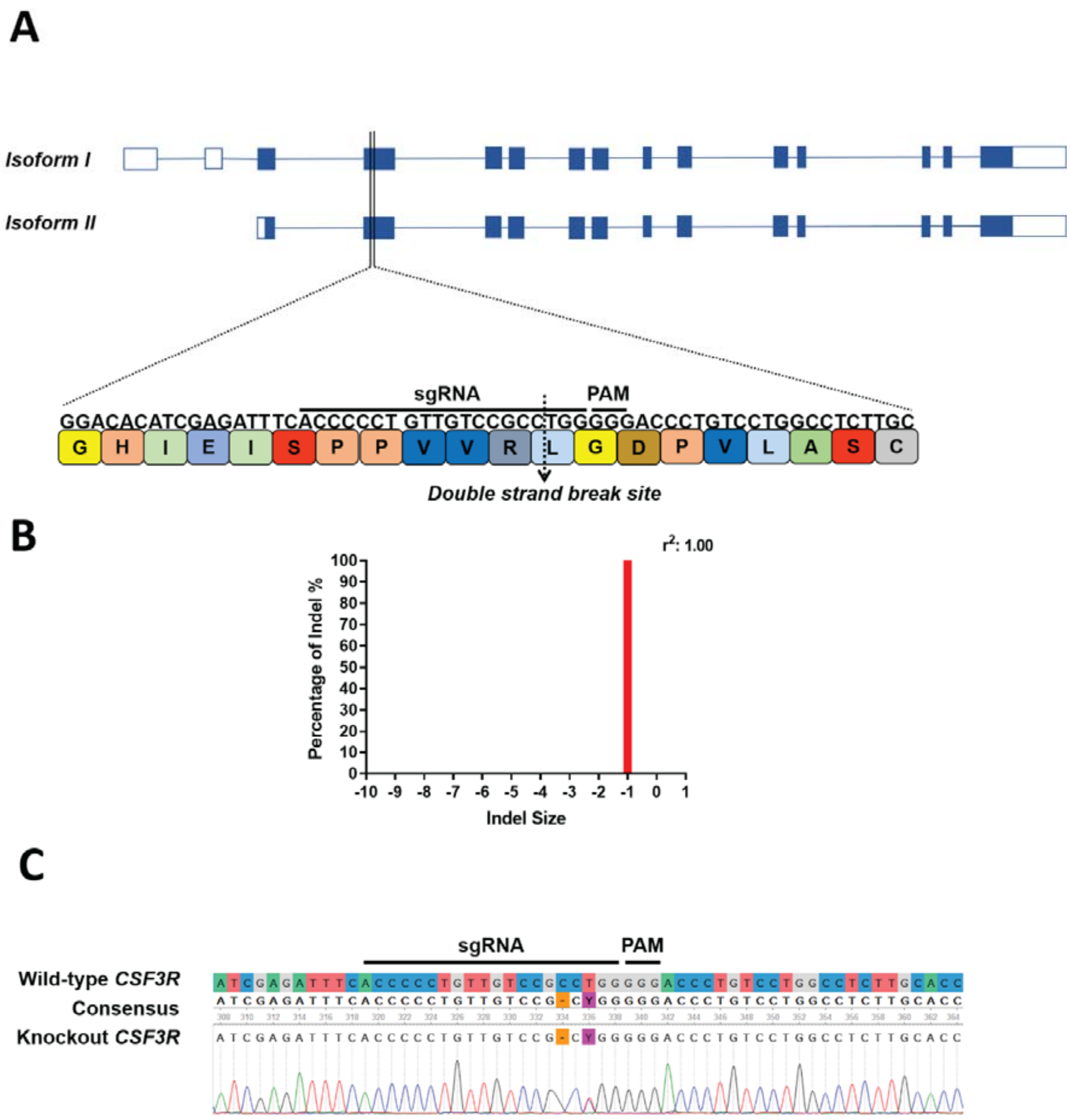

Supplementary Figure 2. Generation of the G-CSFR knock-out NFS-60 cell line. (A) Schematic representation of the CRISPR/Cas9 design strategy to target and knockout two isoforms of G-CSFR (ENSMUSG00000028859). **(B)** Gene editing efficiency in the G-CSFR knock-out NFS-60 cell line assessed by Sanger sequencing and sequence trace decomposition (TIDE). r^2 is calculated to assess the goodness of fit by TIDE algorithm and $r^2 > 0.9$ is considered as a reliable prediction. **(C)** Sequence verification of the G-CSFR knock-out NFS-60 cell line confirms the disruption of the G-CSF receptor gene through a frameshift mutation at the beginning of the ORF.

Supplementary Figure 3

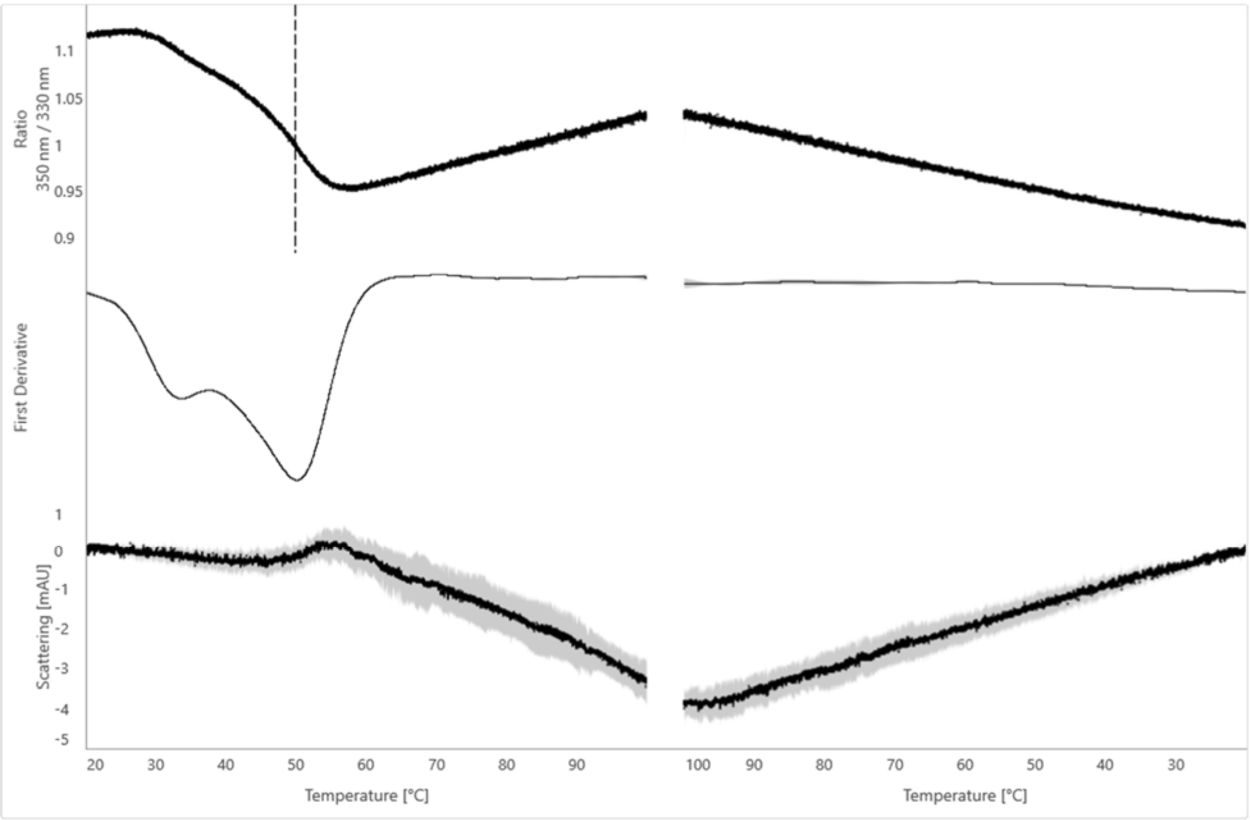

Supplementary Figure 3. DSF melting curve of rhG-CSF in PBS buffer. Upper panes show differential fluorescence change. Middle panes show the first derivative of the latter. Bottom panes show scattering magnitude (left side: temperature ramp up, right side: temperature ramp down), Black lines and gray shades represent average and standard deviations for 5 measurement replicas, respectively.

Supplementary Figure 4

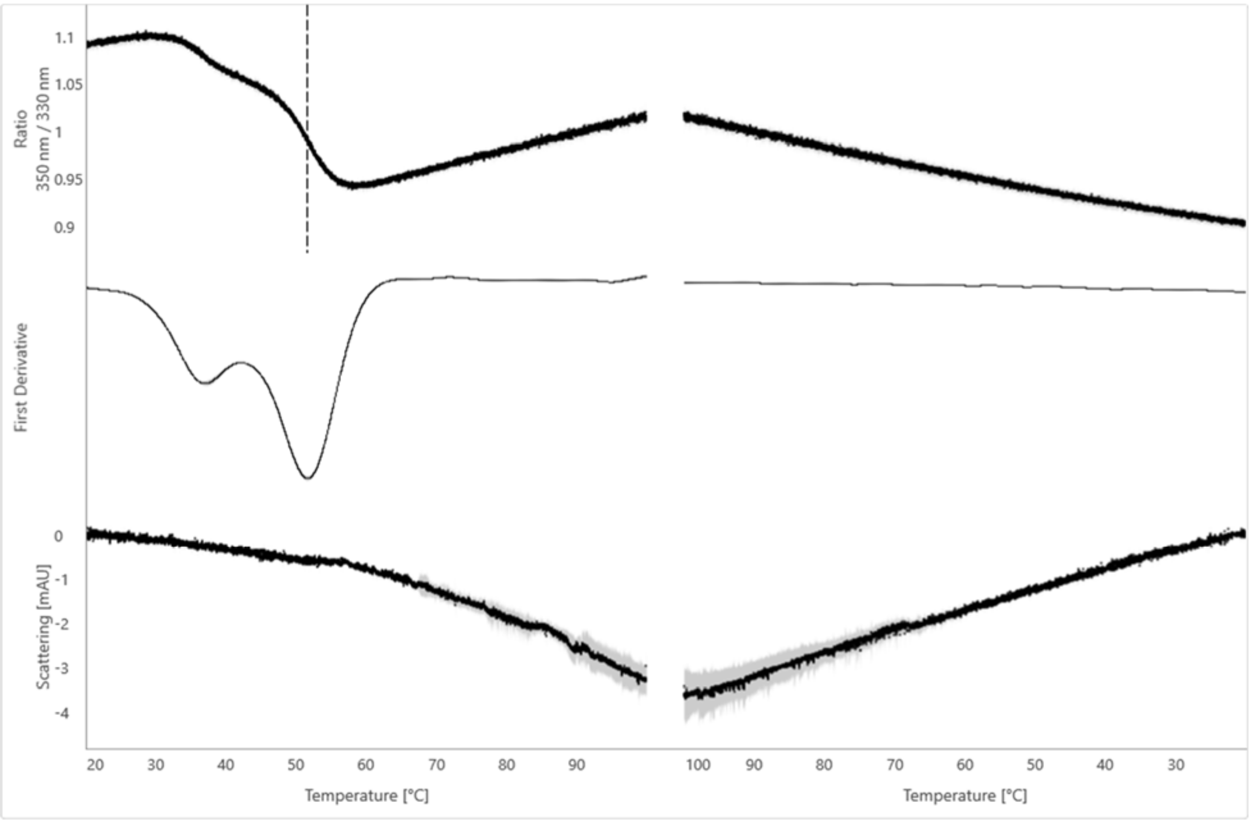

Supplementary Figure 4. DSF melting curve of rhG-CSF in PBS buffer supplemented with 2.5 mM GSH and 0.5 mM GSSG. Upper panes show differential fluorescence change. Middle panes show the first derivative of the latter. Bottom panes show scattering magnitude (left side: temperature ramp up, right side: temperature ramp down), Black lines and gray shades represent average and standard deviations for 5 measurement replicas, respectively.

Supplementary Figure 5

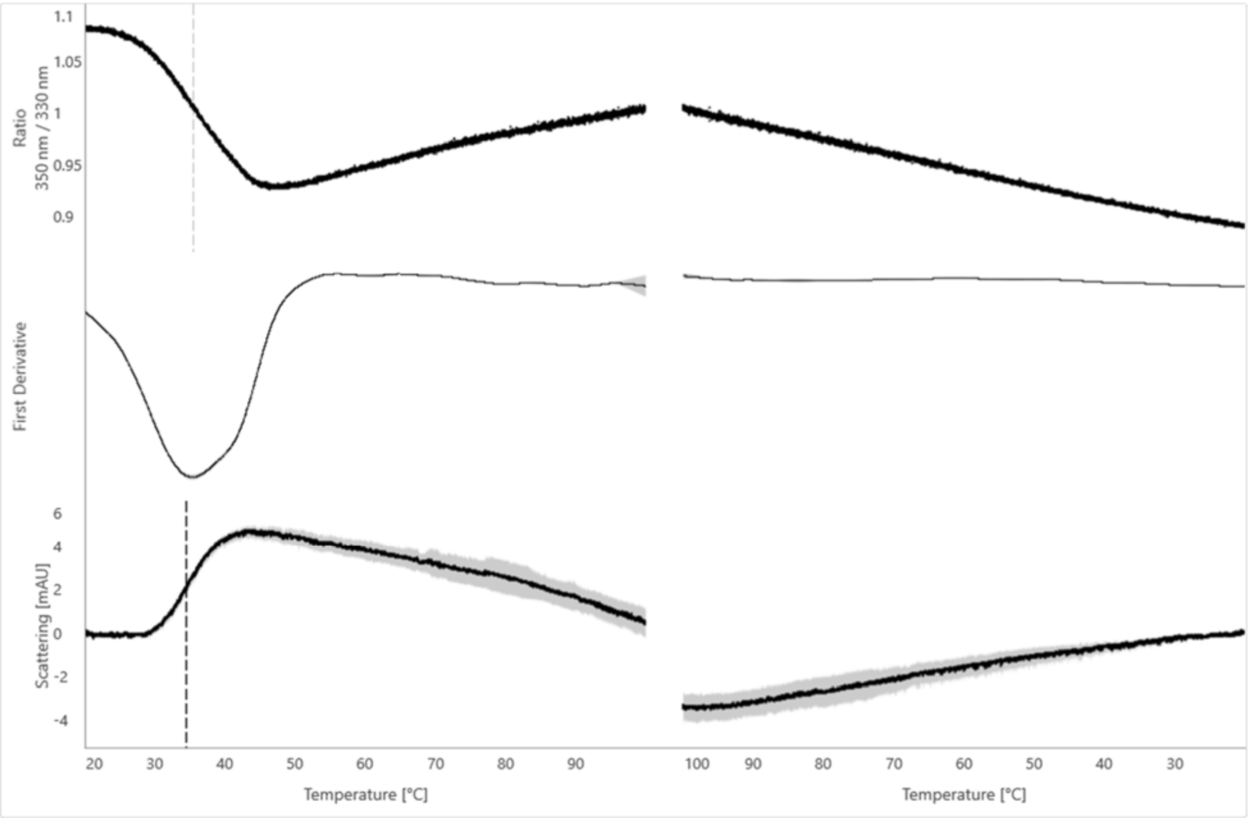

Supplementary Figure 5. DSF melting curve of rhG-CSF in PBS buffer supplemented with 5 mM DTT. Upper panes show differential fluorescence change. Middle panes show the first derivative of the latter. Bottom panes show scattering magnitude (left side: temperature ramp up, right side: temperature ramp down), Black lines and gray shades represent average and standard deviations for 5 measurement replicas, respectively.

Supplementary Figure 6

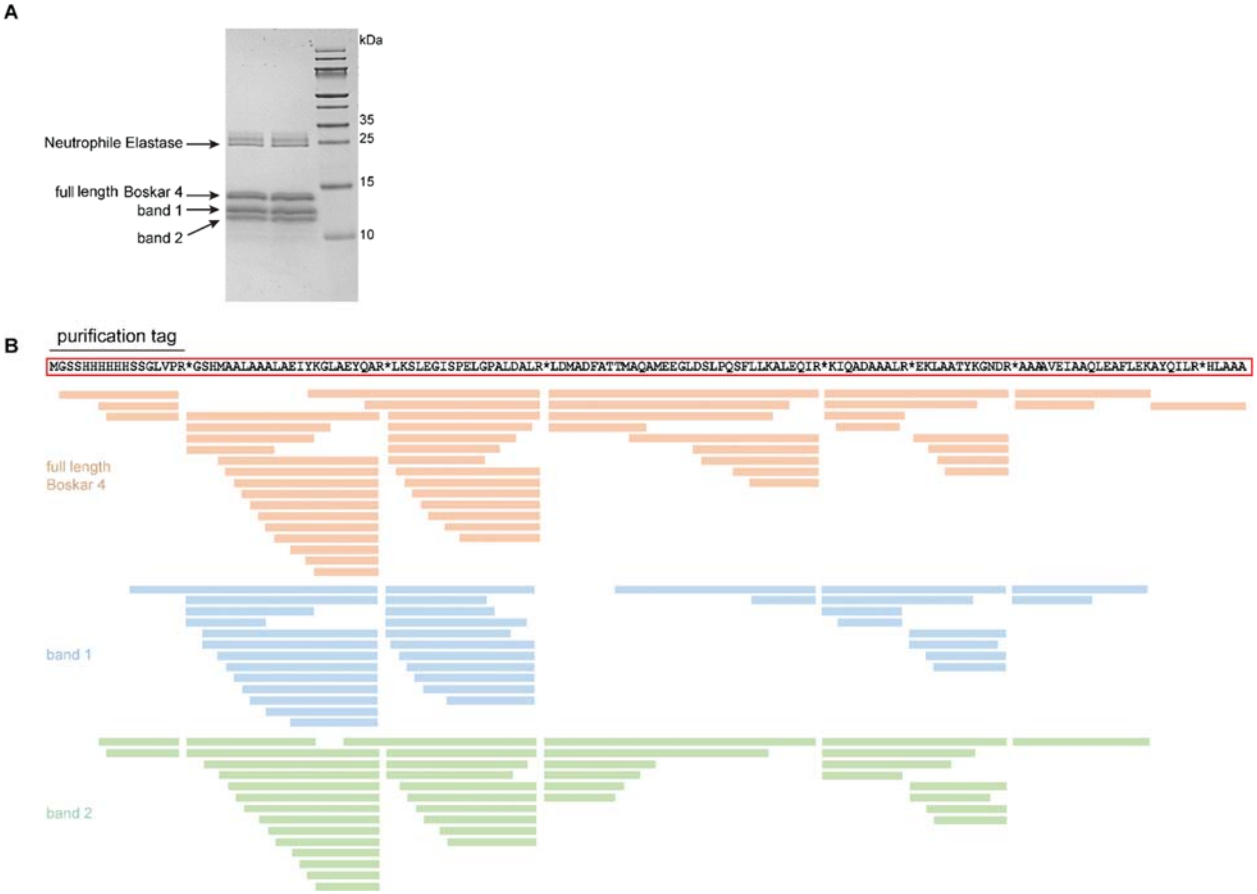

Supplementary Figure 6. Neutrophil elastase partial digestion pattern of Boskar4. (A) SDS-PAGE bands corresponding to full length Boskar4 and proteolytic fragments (bands 1 and 2) were cut and analyzed by mass spectrometry as described (Methods section). **(B)** Schematic view of the sequence of Boskar4 with the peptides that have been identified with a probability of > 99 % (PEP ≤ 0.1) from the different gel bands by mass spectrometry.

Supplementary Figure 7

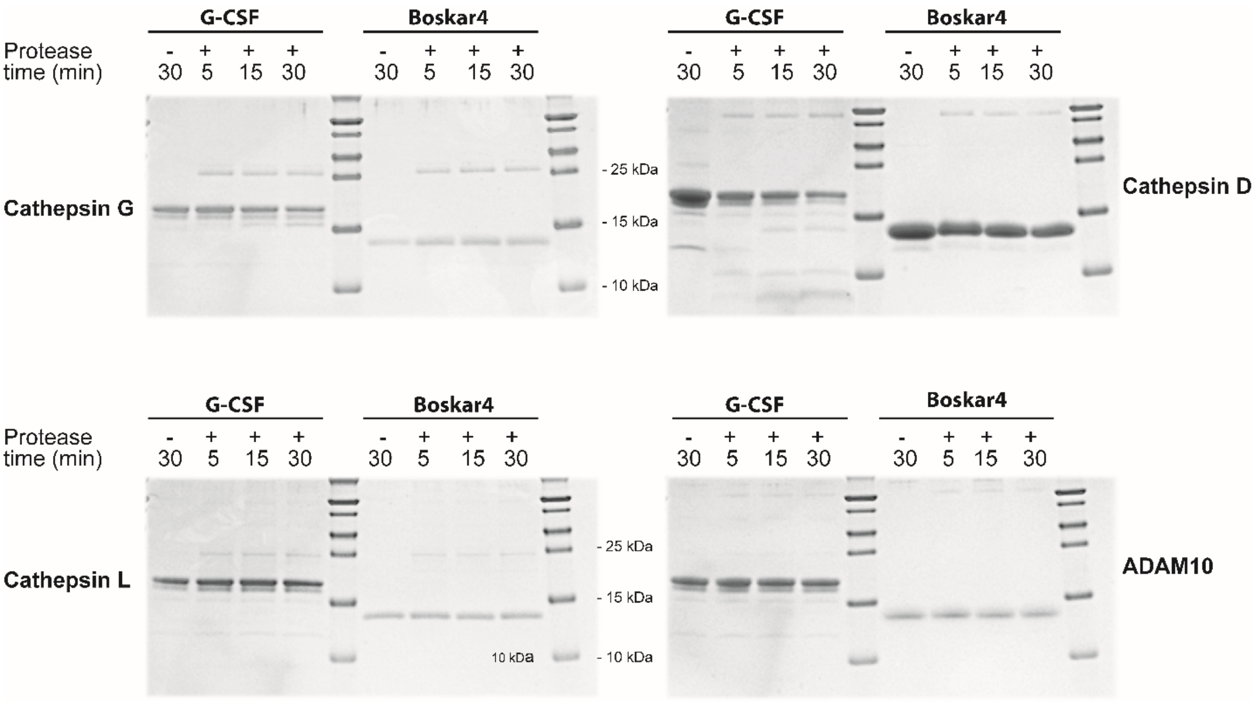

Supplementary Figure 7. Comparison between rhG-CSF and Boskar4 proteolytic stability against a panel of diverse proteases. Both, rhG-CSF or Boskar4 are stable in the presence of ADAM10 and Cathepsin L. However, rhG-CSF is partially degraded by Cathepsin D and Cathepsin G, whereas Boskar4 appears to be resistant. The molecular marker used is the same in all gels, and the molecular weight of the relevant standard bands are indicated in the middle.

Supplementary Figure 8

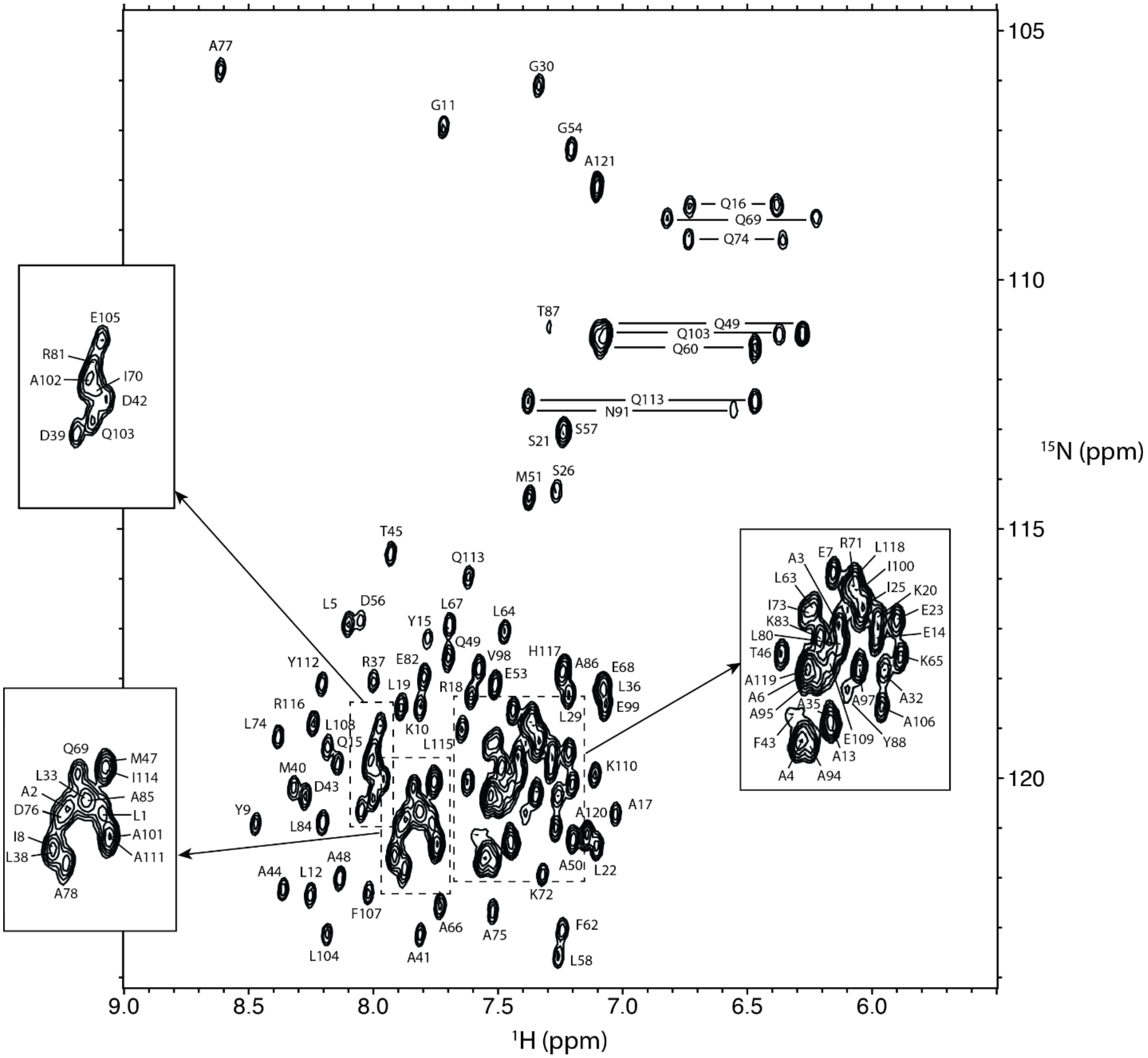

Supplementary Figure 8. ^{1}H-^{15}N HSQC spectrum of Boskar4. Backbone and side chain amides assignments are indicated. Inset shows close-up views of the resonance assignments in packed regions. Signals of A77 and A121 are folded.

Supplementary Figure 9

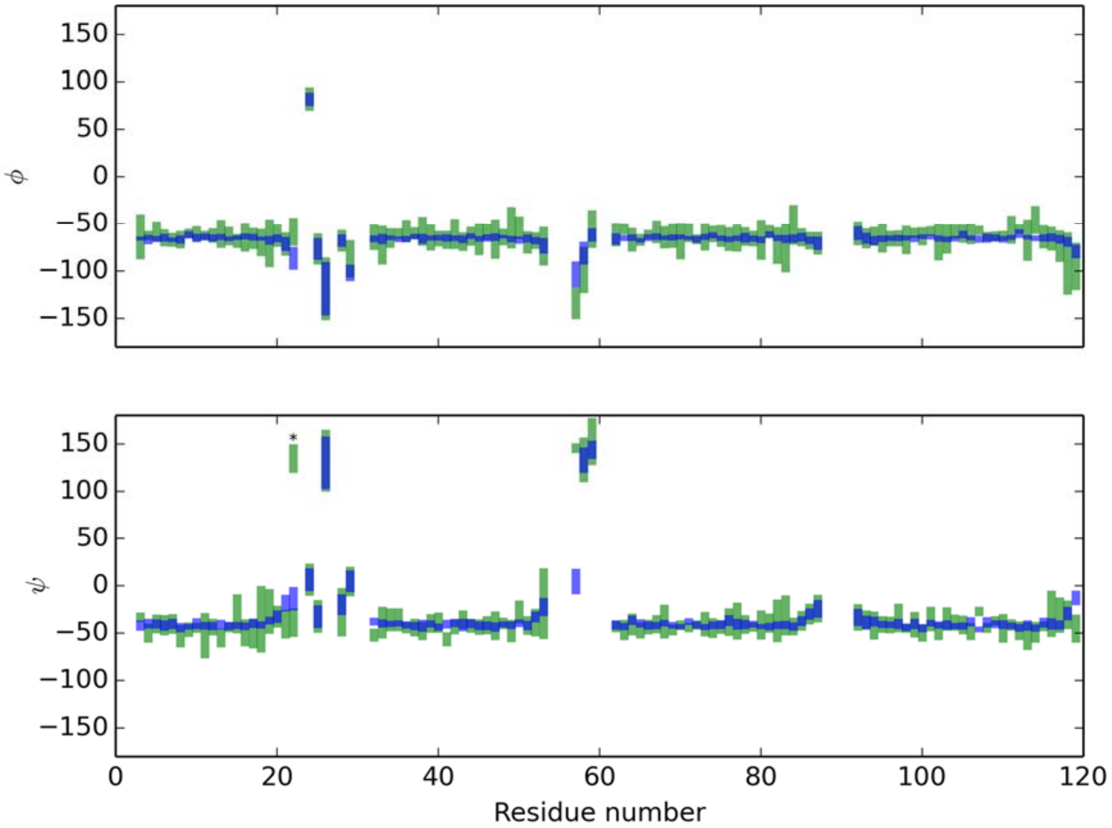

Supplementary Figure 9. Agreement between CoMAND backbone angles and chemical shift-based predictions of Boskar4. Backbone φ and ψ angles (top and bottom panels, respectively) determined via CoMAND greedy optimisation for single residues are plotted as green bars. The height of the bars represents the extent of the applied restraint, with a minimum of 6°. The polymorphism in the L22 ψ angle is marked with an asterisk. Also plotted are the high-confidence predictions based on chemical shift comparisons using TALOS-N [81] with their calculated error ranges (blue bars). The two methods show very good agreement, with only one angle (57 ψ in the α2-α3 loop) showing major disagreement.

Supplementary Figure 10

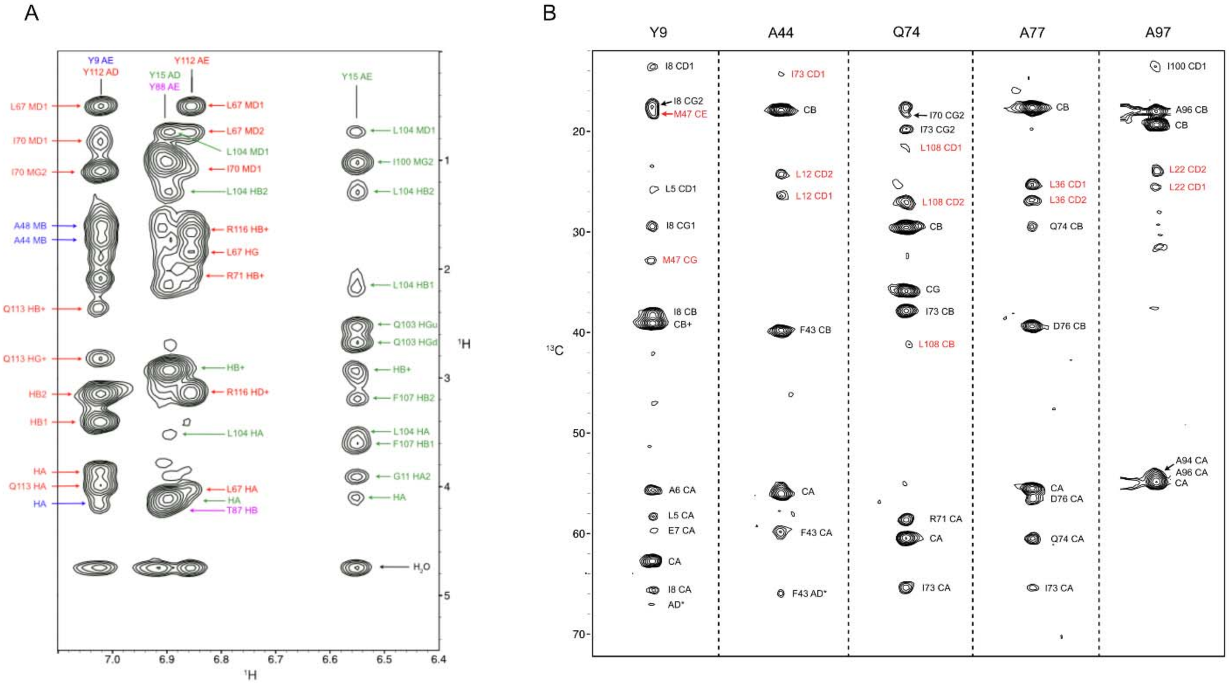

Supplementary Figure 10. Inter-helical contacts in Boskar4. A) A detail of the 2D 1H-NOESY spectrum of Boskar4 acquired with a destructive filter on 15N-bound protons to allow clear observation of contacts to aromatic protons. The selected region shows a subset of cross-peaks between aromatic and aliphatic protons. Selected contacts are annotated, coloured according to the aromatic proton involved. The proton naming convention is: A (degenerate aromatic), M (methyl), + (degenerate methylene). Non-degenerate methylene groups that have not been stereospecifically assigned are labelled u (upfield) and d (downfield). Intra-residue contacts are labelled by the atom name only. Unlabelled peaks have significant contributions from multiple mostly short- or medium-range contacts. A number of long-range contacts can be identified to define the Boskar4 four-helix bundle. **B)** Strips extracted from the 3D-CNH-NOESY of Boskar4. Cross-peaks are annotated as in panel A, except that colouring now identifies inter-helical contacts (red). Folded aromatic resonances are marked with an asterisk. Cross-peaks to neighbouring strips have been removed for clarity. The selected strips are from residues in all four helices.

Supplementary Figure 11

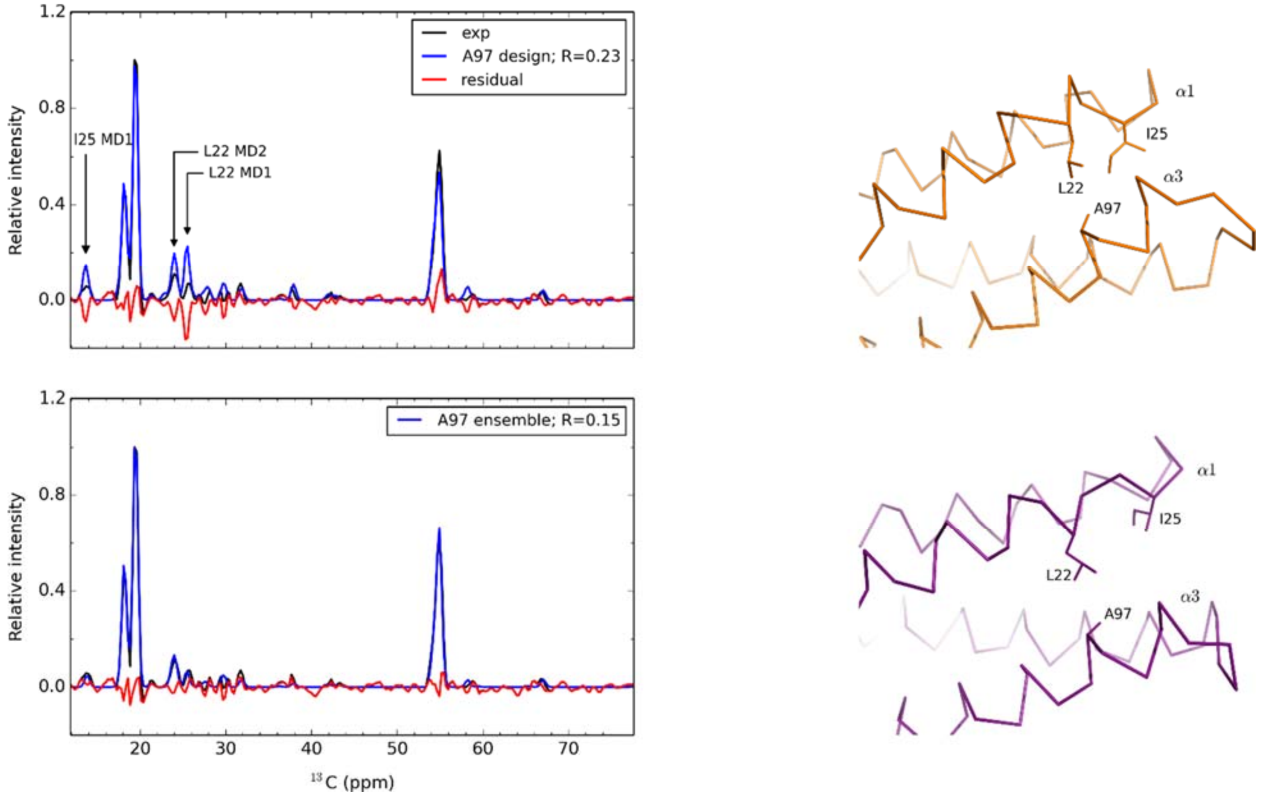

Supplementary Figure 11. The Boskar4 solution structure differs from the design model in the α1-α3 interface. R-factor calculations for A97 at the C-terminus of α3 identify three contacts that have over-estimated intensities in the design model (top panel). These involve methyl groups of L22 and I25 at the C-terminus of α1. Selection of models to optimise R-factors results in very well reproduced intensities for these contacts and a more open bundle than anticipated in the design. Note that of the three contacts indicated, only the $C^{\delta 1}$ methyl (MD1) of L22 represents a high proportion of the intensity in the back-calculated spectrum for the ensemble. The others are either largely explained by local contacts on α3 (I25 MD1) or by a mixture of several contacts (L22 MD2). This demonstrates that R-factor optimisation is driven as much by the absence of peaks as their presence.

Supplementary Figure 12

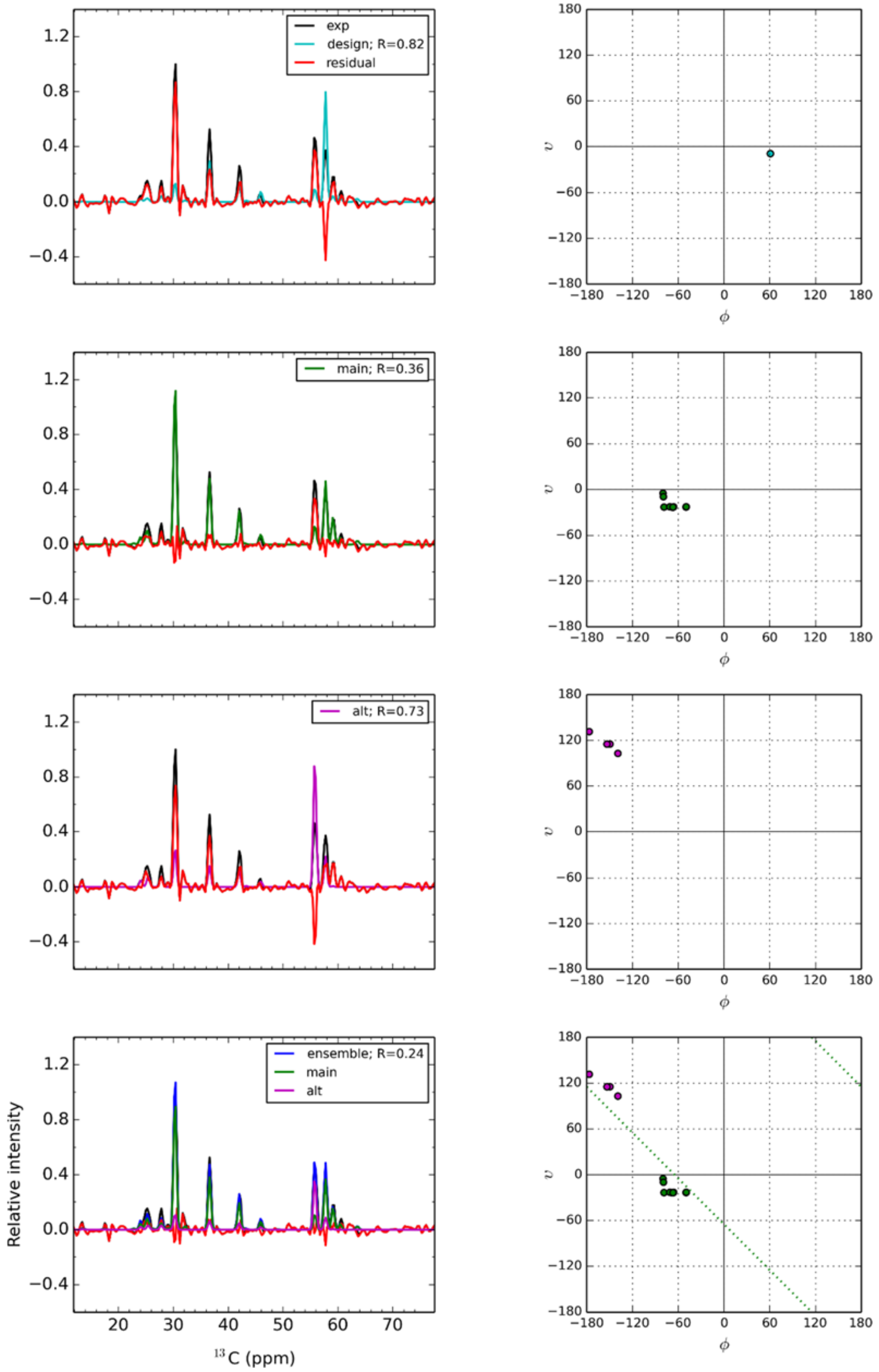

Supplementary Figure 12. Boskar4 shows a backbone polymorphism in the α1 helix cap. The design model contains a highly unusual sequence of three consecutive positive φ angles over residues E23-I25 that were inherited from the parent crystal structure. This conformation is, however, incompatible with the CNH-NOESY data (top row). Single-residue R-factor optimisation provided canonical helical angles for residue L22 and E23. However, it also shows that the strip for E23 is not well reproduced by a single conformation (second row). Factorisation calculations indicate a second conformation involving a polymorphism at the L22 ψ angle involving 20-25% of the population (third row). Accordingly, rebuilding the region to accommodate this polymorphism for 4 of the 17 final models reproduces intensities for the E23 strip very well (bottom row). The panels on the left show the distribution of backbone dihedrals for E23. Note that CoMAND employs a shifted Ramachandran space, whereby the φ angle of each residue is correlated with the ψ angle of the previous residue [33]. In this context we refer to ψ_{i-1} as υ_i, and the plots in this figure are plotted in this manner. In this shifted space, conformations that lie along anti-diagonal lines of the form φ=- ψ+θ share a common chain path defined by θ and are related by rotation of the plane of the peptide bond. One such line, for θ=-65°, is plotted in the bottom panel (dotted green line) and links the two conformers for E23. Thus, a motional model for the α1 helix cap involves a ~120° flipping of the L22-E23 peptide bond with a small change in the overall chain path. This is accommodated by conformational variability along the α1-α2 loop, particularly at S26. Notably, chemical shift-based dihedral angle predictions using TALOS failed to provide a high-confidence prediction for E23, but predicted canonical helical angles for L22. This demonstrates the high level of local detail that CoMAND analysis can provide.

210

Supplementary Figure 13

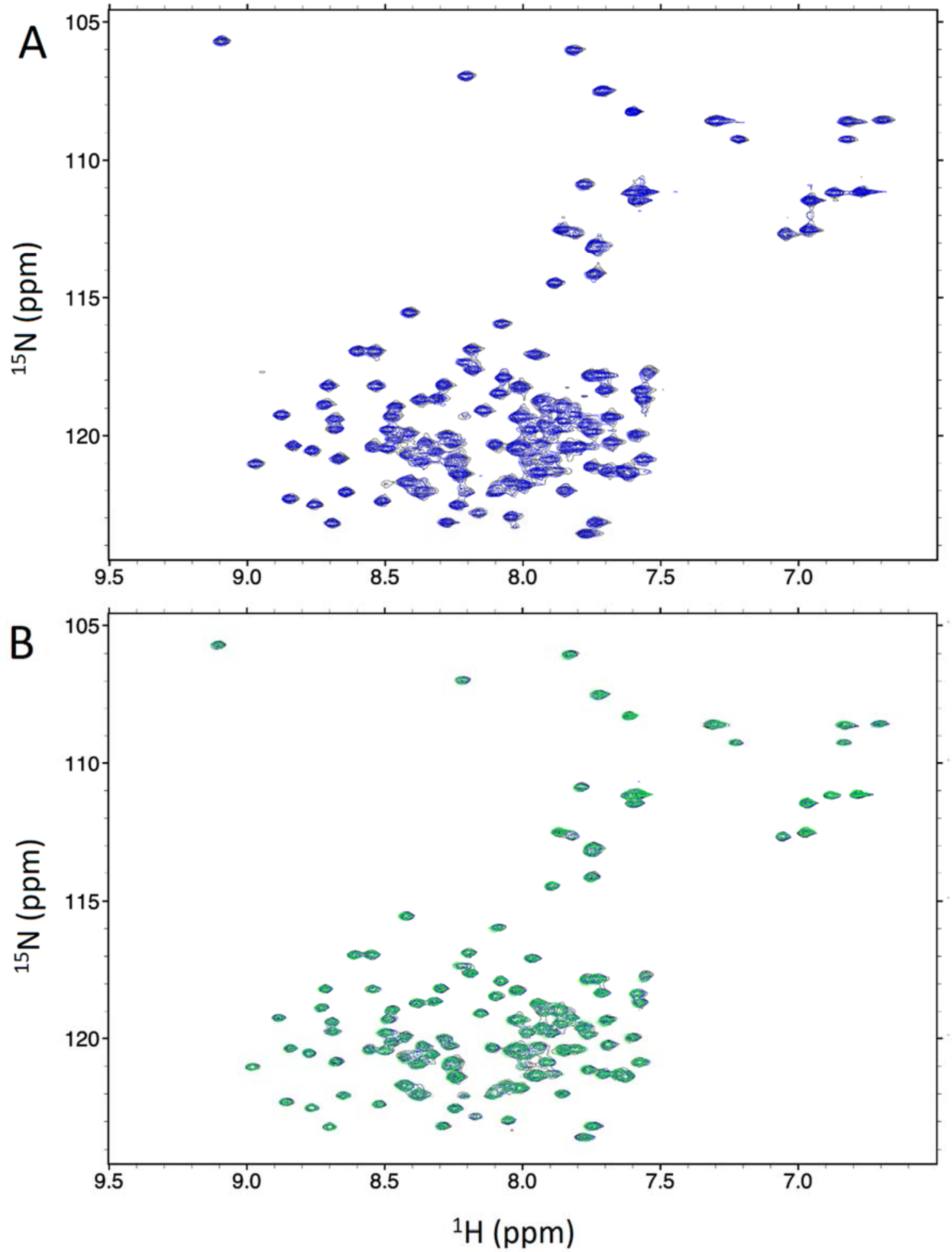

211

212

Supplementary Figure 13. NMR samples of Boskar4 remain constant after concentration and temperature ramps. A) Overlays of 15N HSQC spectra (313 K) of Boskar4 are shown, with NS=4 for concentrated and NS=8 for diluted samples. A) Comparison of a sample at a concentration comparable to that used for structure determination (~800 μM; black) and after a four-fold dilution (~200 μM; blue). The spectrum of the diluted sample has been scaled by a factor of 2 relative to the reference. **B)** Comparison of the dilute sample from panel A before (black) and after (green) heat treatment via a 1 degree per minute temperature ramp from 25 to 100 ºC and back. Both spectra are plotted at the same contour level. No significant changes are observed in the spectra, indicating that the extent of any specific dimerisation interaction does not change across this concentration range, nor is induced by the heat treatment.

Supplementary Figure 14

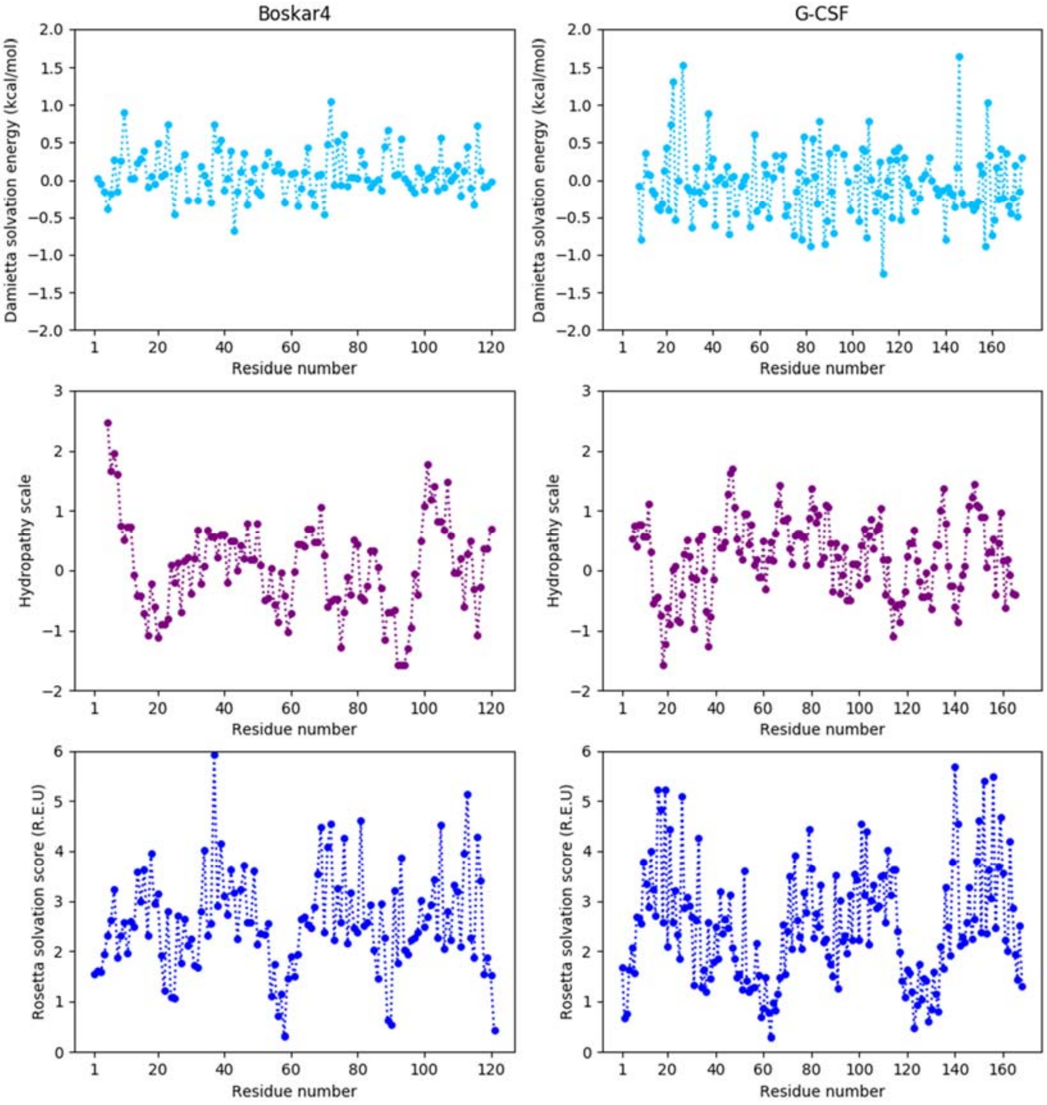

Supplementary Figure 14. Calculated solvation properties of G-CSF and Boskar4 structures. Damietta solvation energy term (top), Kyte-Doolittle sequence hydropathy scale (middle), and the Rosetta solvation score (bottom); See Materials and Methods.

Supplementary Figure 15

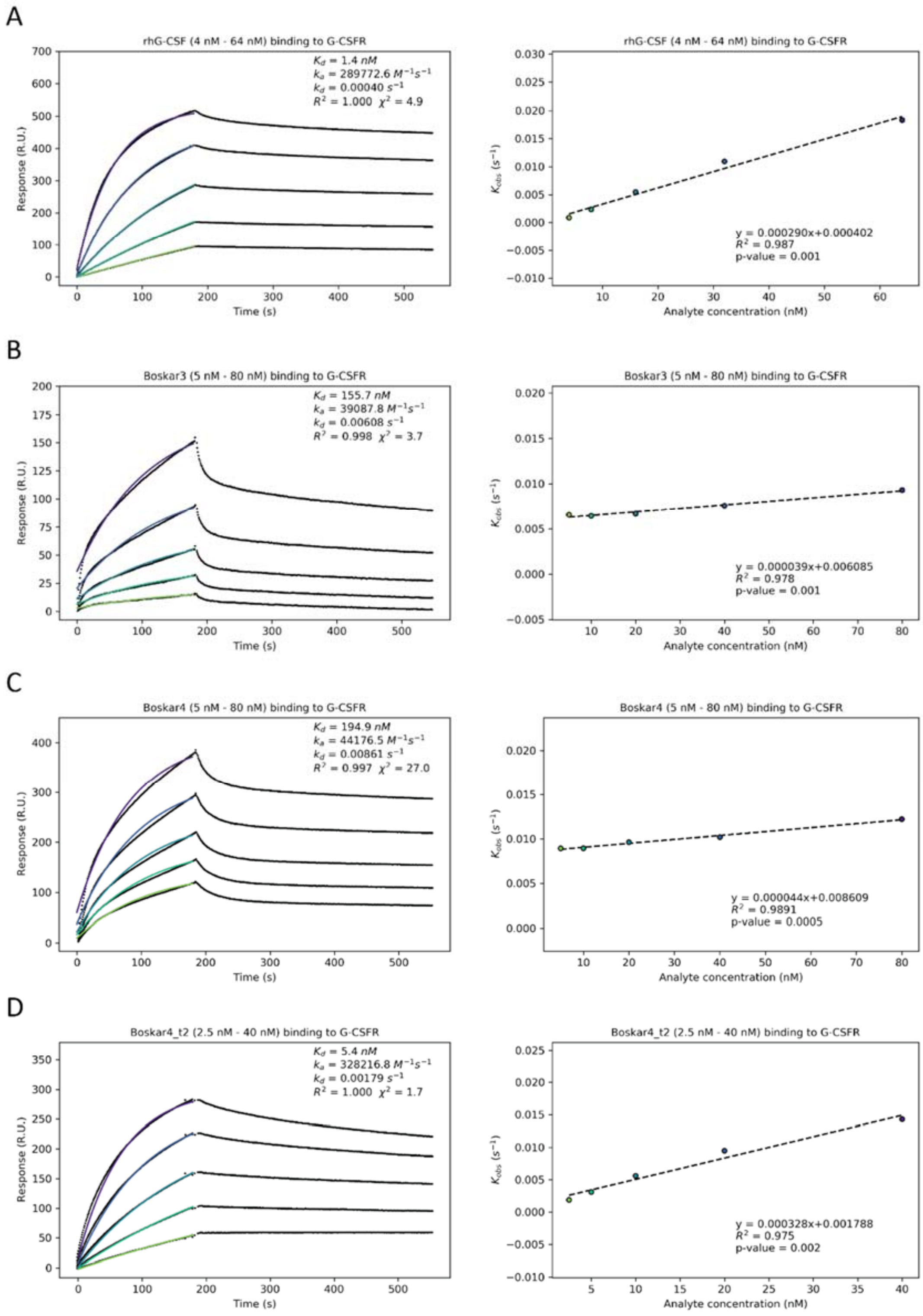

Supplementary Figure 15. SPR sensorgrams of (A) rhG-CSF, (B) Boskar3, (C) Boskar4, and (D) Boskar4_t2 binding to rhG-CSFR and their binding kinetics fit. Sensograms and association phase fits are shown (left-side panes; data points: black dots, fits: cyan-to-purple curves) against their respective k_{obs} fits (Table 1). rhG-CSF, recombinant human G-CSF; rhG-CSFR, recombinant human G-CSF receptor; SPR, surface plasmon resonance. The linear regression coefficients of determination (R^2) and p-values for the correlation between k_{obs} and analyte concentration.

Supplementary Figure 16

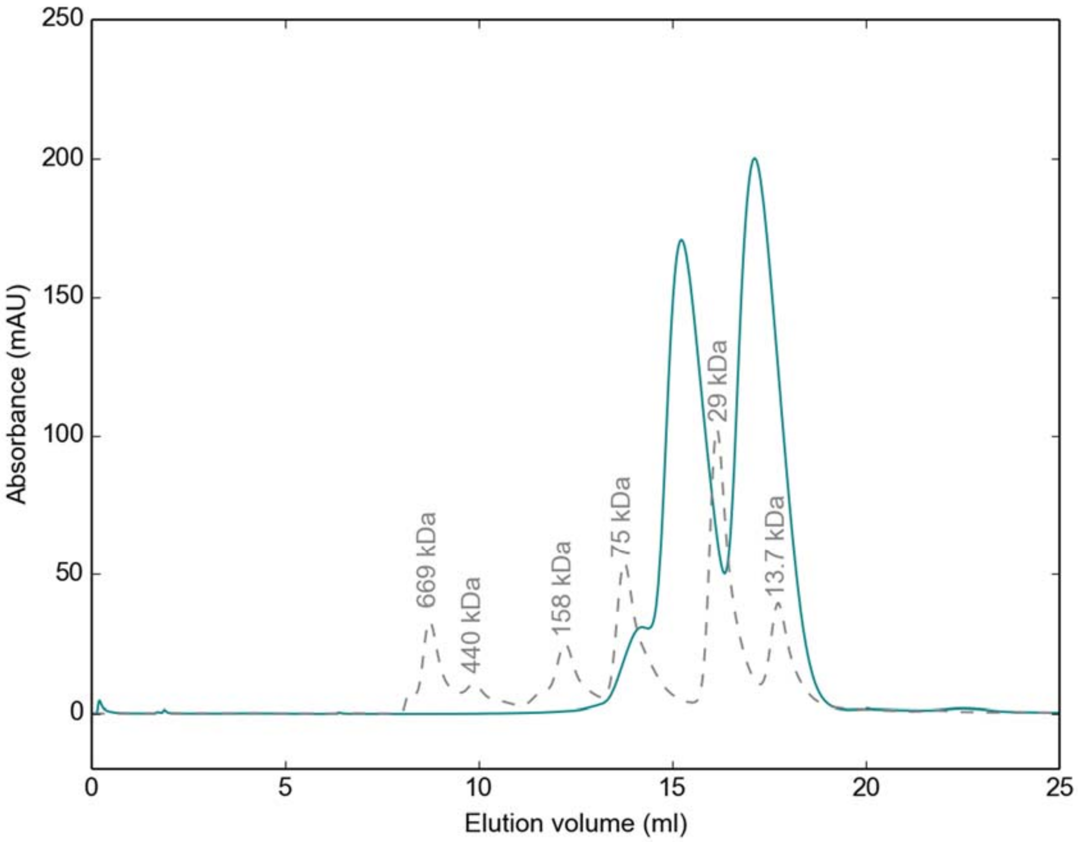

Supplementary Figure 16. Analytical size-exclusion elution profile of Boskar3 (teal) shows almost equipartition between monomeric and dimeric species. Calibration curve shown in grey.

Supplementary Figure 17

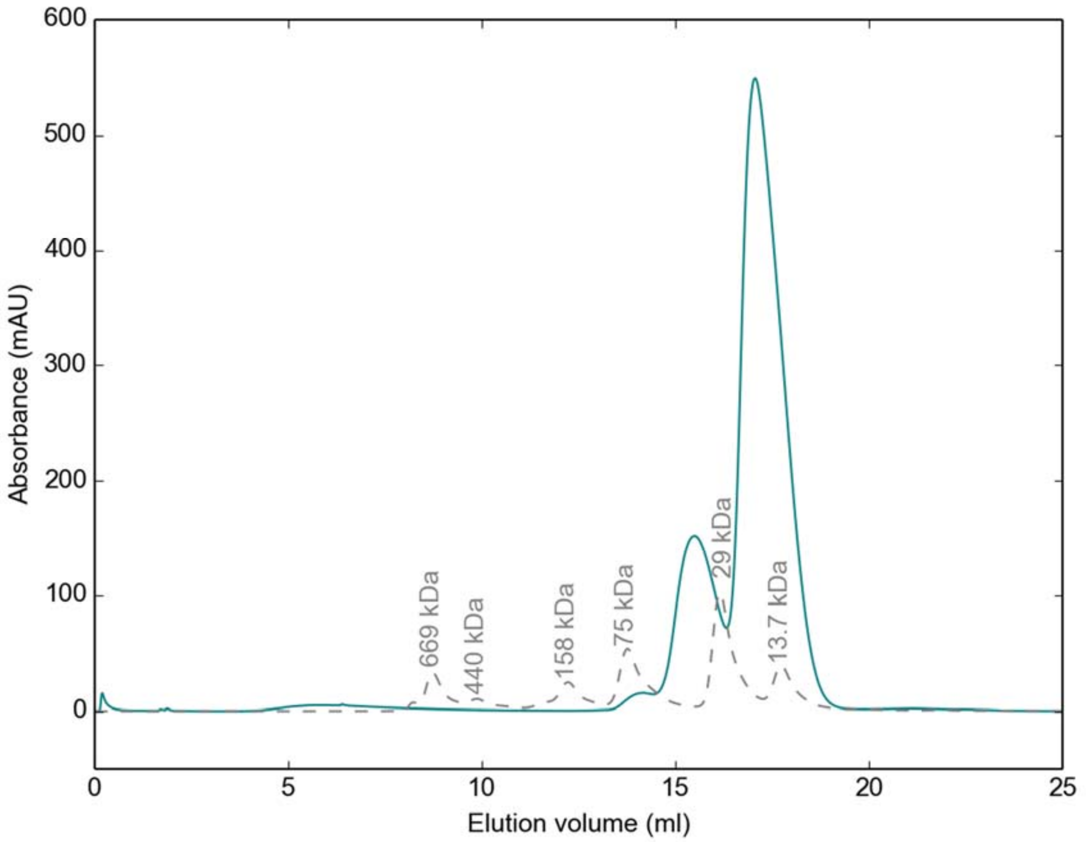

Supplementary Figure 17. Analytical size-exclusion elution profile of Boskar4 (teal) shows dimeric (minor) and monomeric (major) species. Calibration curve shown in grey.

Supplementary Figure 18

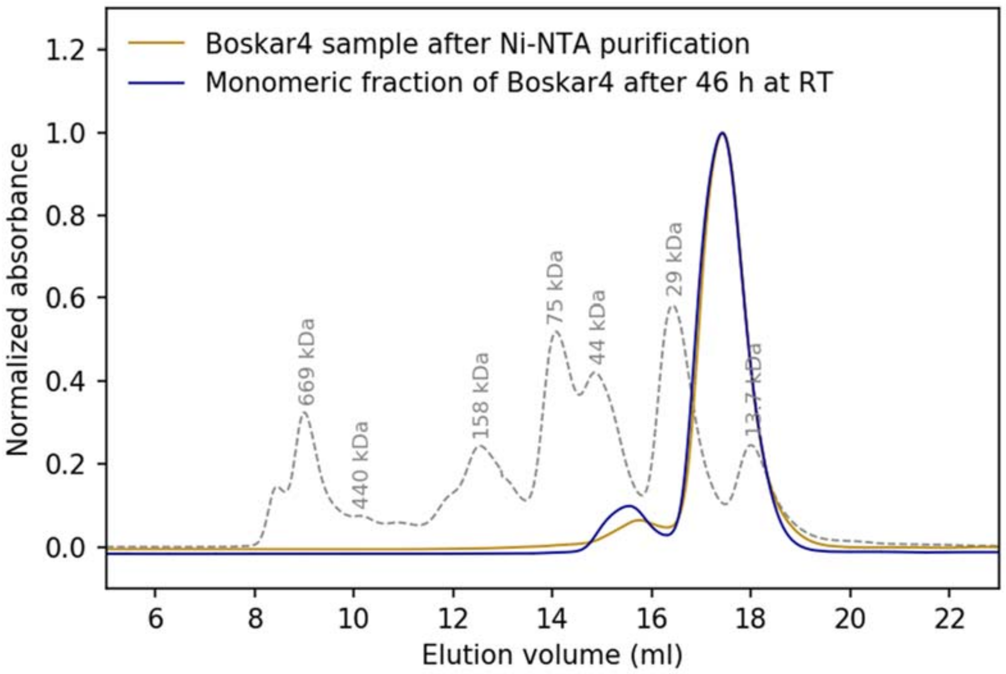

Supplementary Figure 18. Analytical size-exclusion elution profile of Boskar4 (brown) after affinity purification, and after isolation and 46-hour incubation of the monomeric fraction at room temperature (blue). Calibration curve shown in grey.

Supplementary Figure 19

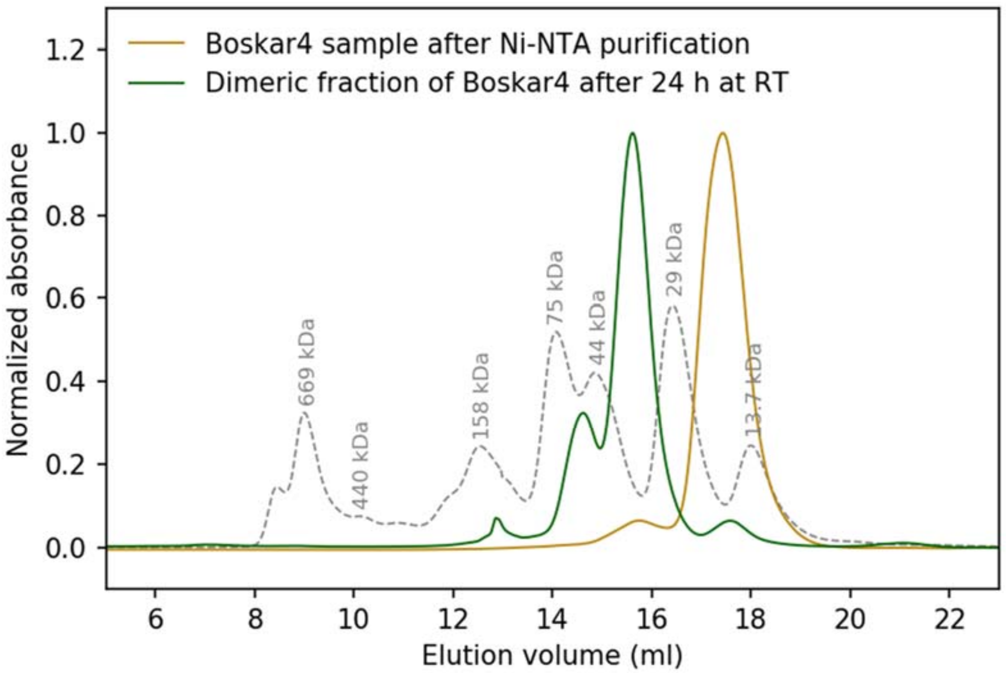

Supplementary Figure 19. Analytical size-exclusion elution profile of Boskar4 (brown) after affinity purification, and after isolation and 24-hour incubation of the dimeric fraction at room temperature (green). Calibration curve shown in grey.

Supplementary Figure 20

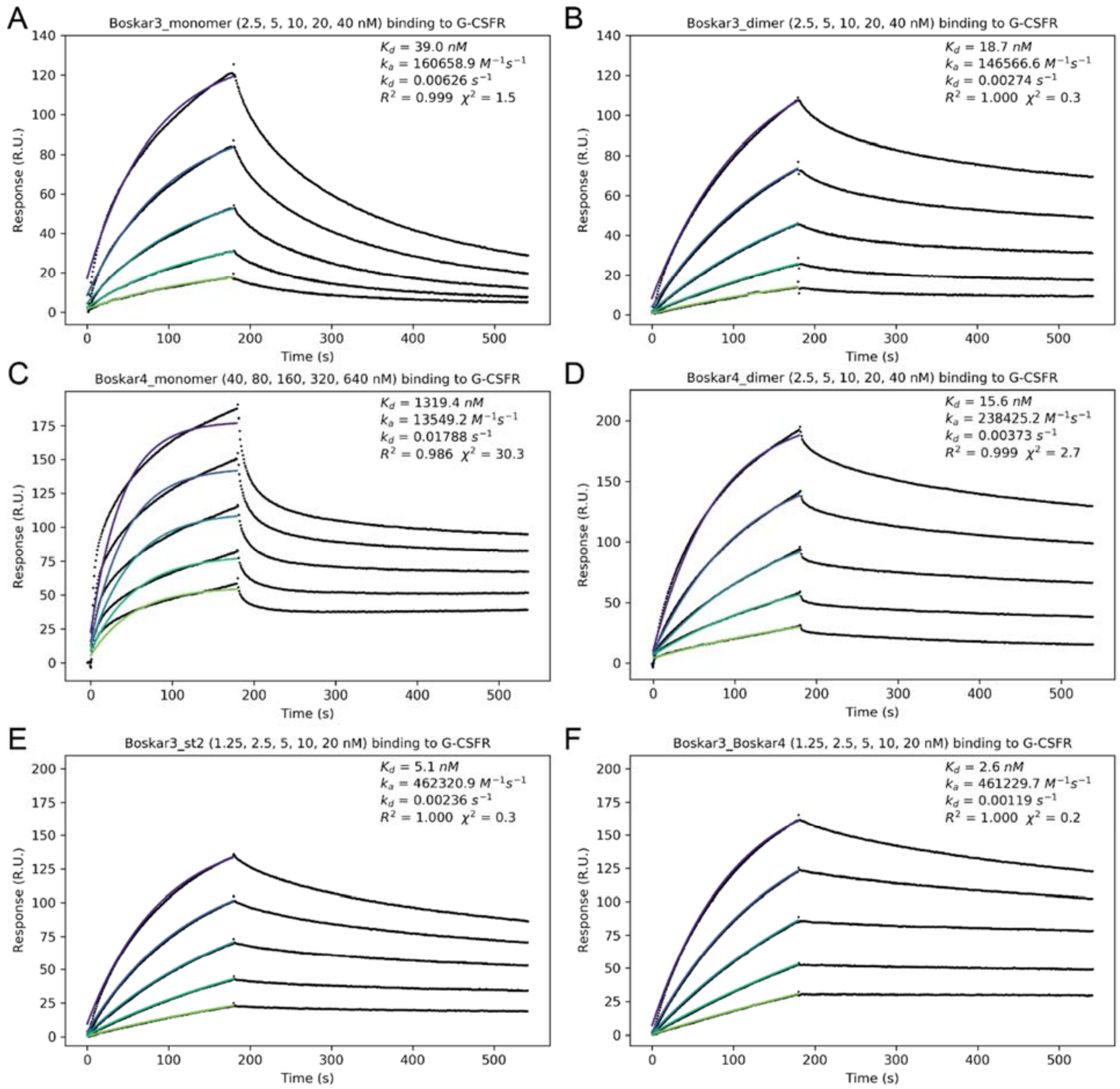

Supplementary Figure 20. SPR binding experiments show a stronger binding pattern of bivalent designs towards rhG-CSFR. (A, B) The dimeric species of Boskar3 binds tighter ($K_D \approx 18.7$ nM) than the monomeric species ($K_D \approx 39$ nM). **(C, D)** The dimeric species of Boskar4 binds tighter (KD ≈ 15.6 nM) than the monomeric species ($K_D \approx 1319$ nM). **(E)** The short-linker tandem of Boskar3 (Boskar3_st2) binds better than Boskar3 fractions ($K_D \approx 5.1$ nM). **(F)** The short-linker tandem of Boskar3 and Boskar4 domains (Boskar3_Boskar4) binds comparably well to other short-linker bivalent constructs ($K_D \approx 2.6$ nM).

Supplementary Figure 21

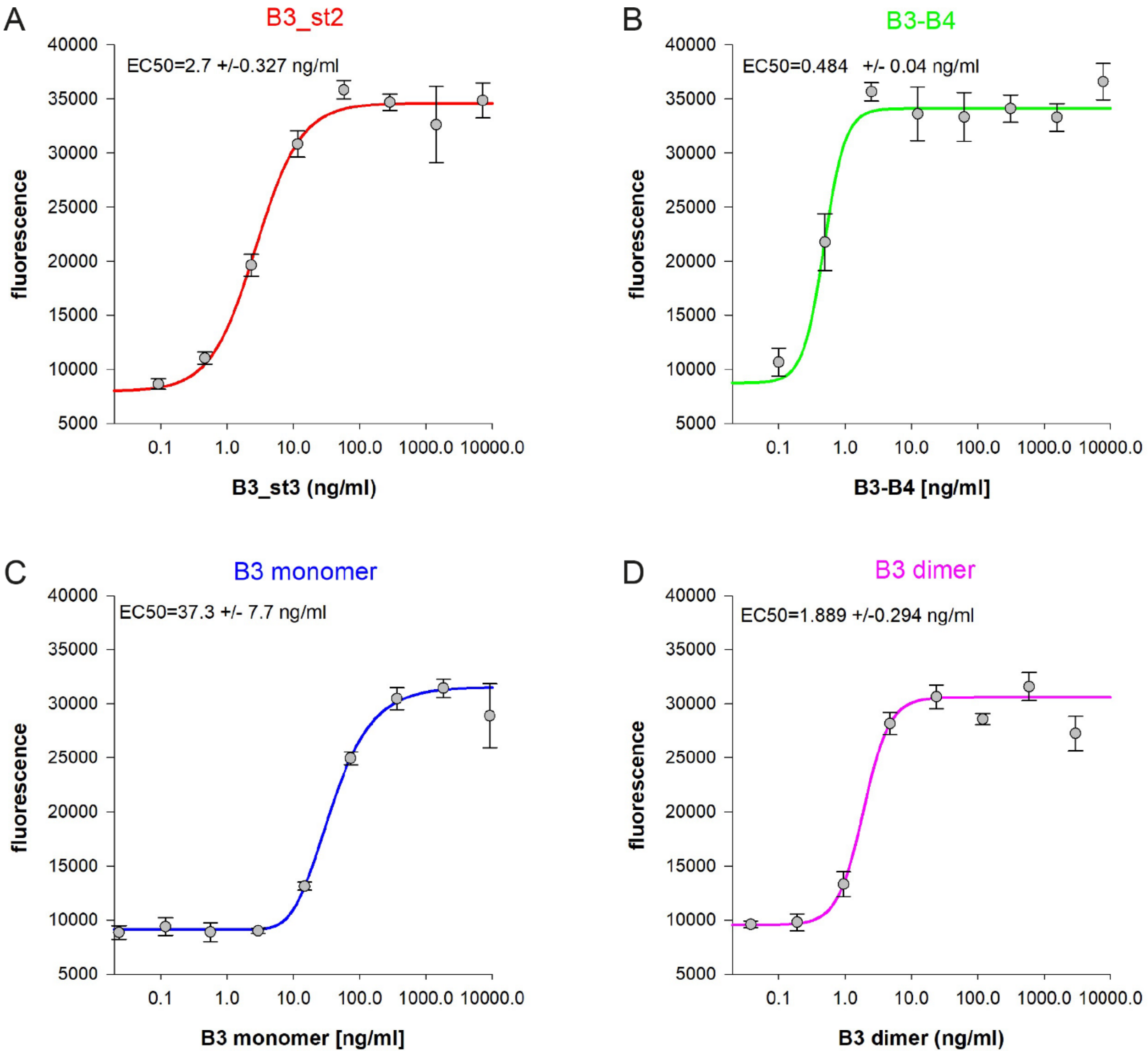

Supplementary Figure 21. Proliferative activity of bivalent designs is stronger than monovalent factions in NFS-60 cells. (A) Boskar3_st2, **(B)** Boskar3_Boskar4, and **(D)** the dimeric Boskar3 fraction all have lower proliferative EC50 values than **(C)** monomeric Boskar3. Data show mean ± standard deviation across 3 biologically independent replicates.

Supplementary Figure 22

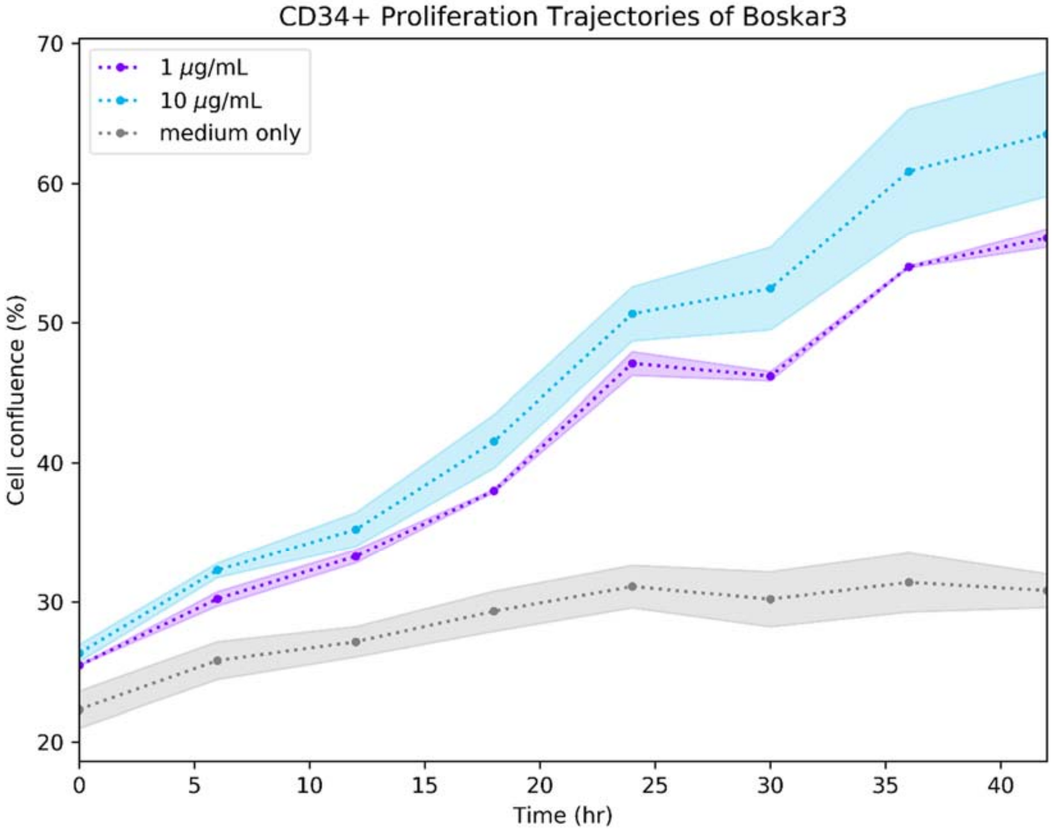

Supplementary Figure 22. Boskar3 induces HSPCs proliferation. Time-response curves of primary human CD34+ cells proliferation with Boskar3 treatment, using two different concentrations. Datapoints and shades indicate mean ± standard deviation across 3 biologically independent replicates.

Supplementary Figure 23

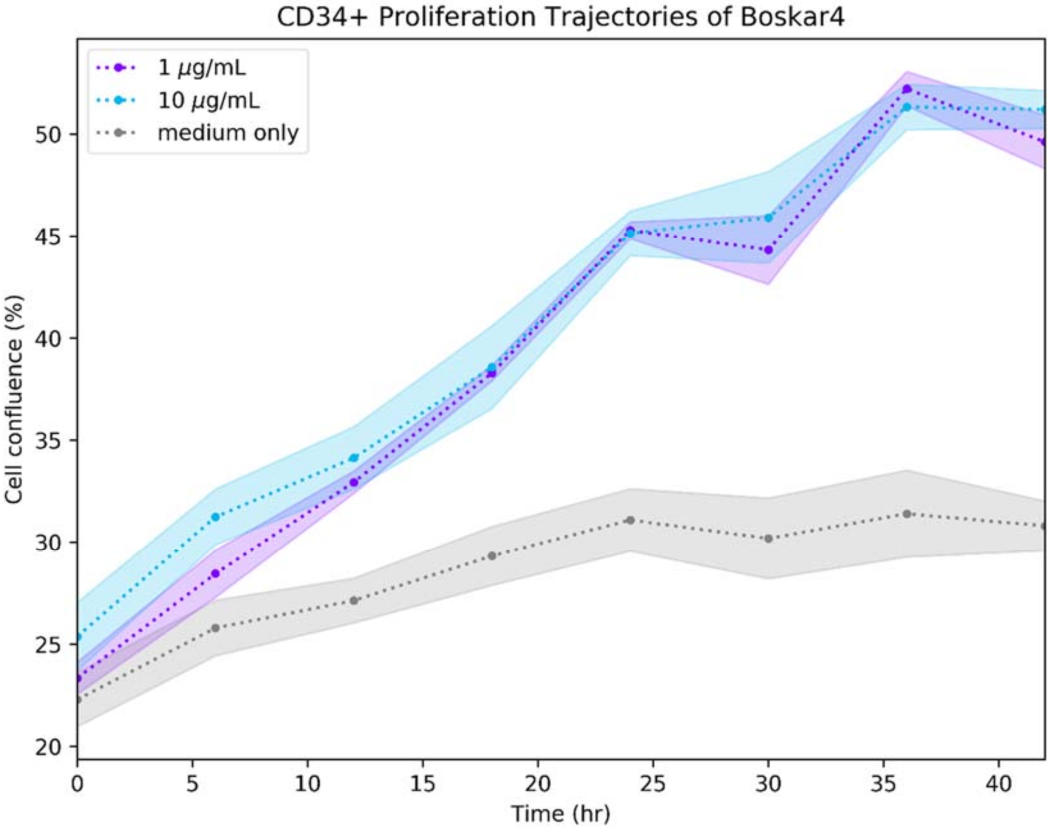

Supplementary Figure 23. Boskar4 induces HSPCs proliferation. Time-response curves of primary human CD34+ cells proliferation with Boskar4 treatment, using two different concentrations. Datapoints and shades indicate mean ± standard deviation across 3 biologically independent replicates.

Supplementary Figure 24

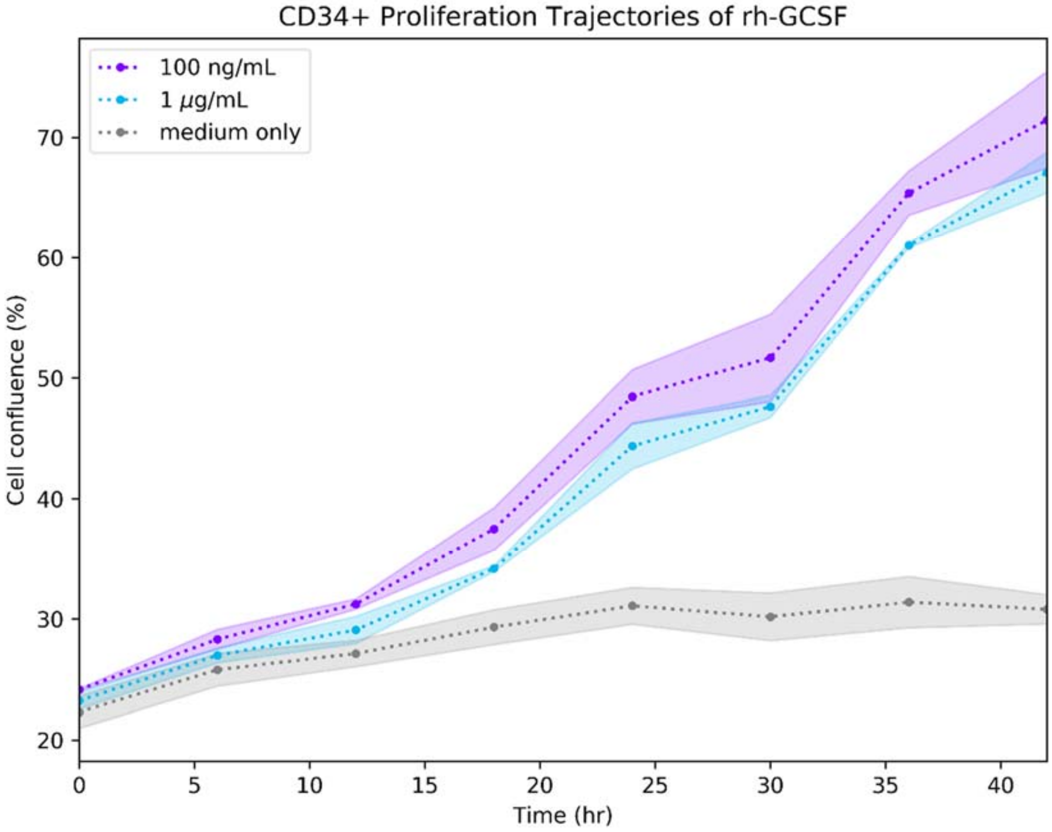

Supplementary Figure 24. rhG-CSF induction of HSPCs proliferation. Time-response curves of primary human CD34+ cells proliferation with rhG-CSF treatment, using two different concentrations. Datapoints and shades indicate mean ± standard deviation across 3 biologically independent replicates.

Supplementary Figure 25

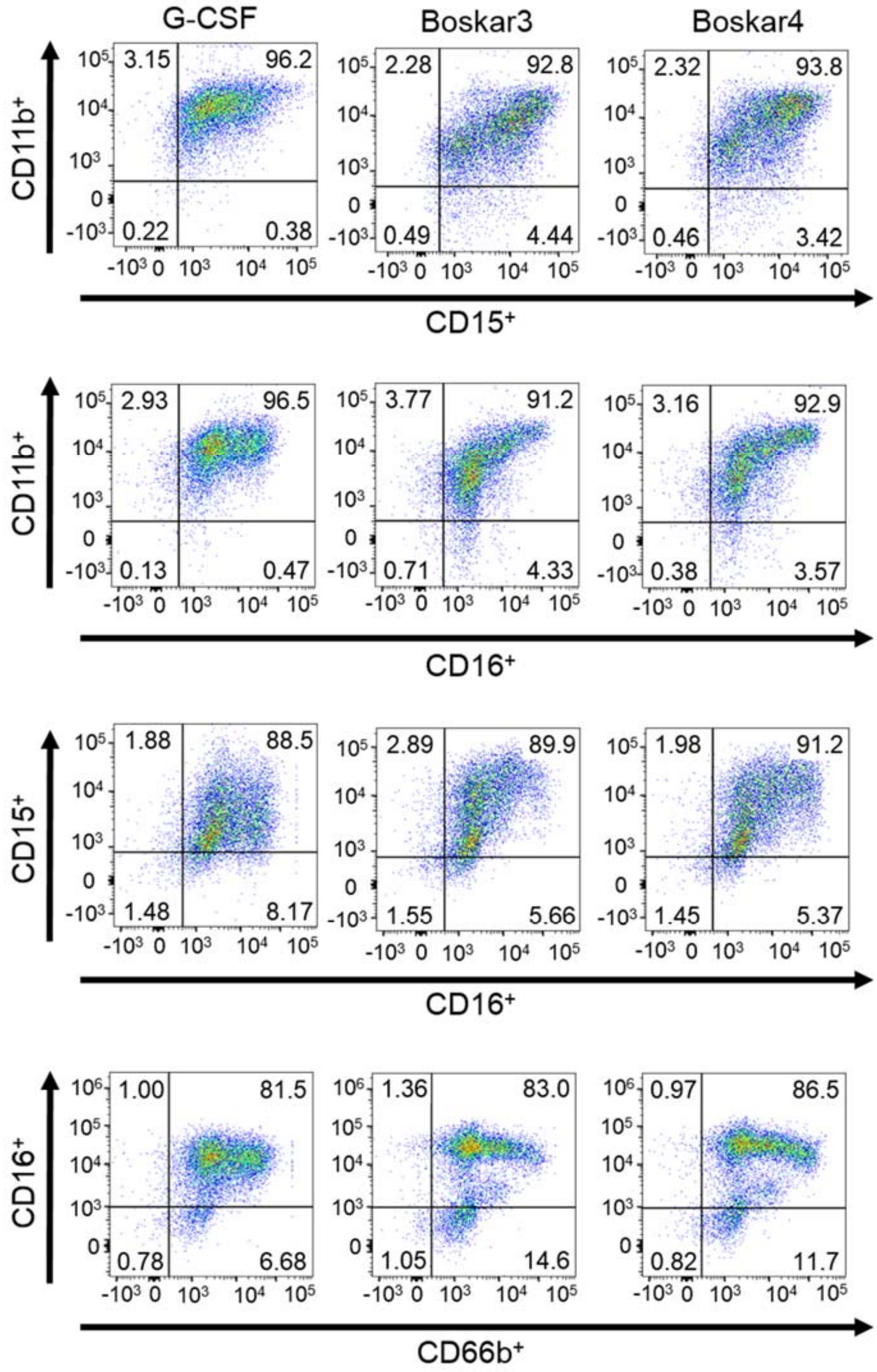

Supplementary Figure 25. Representative FACS profiles of neutrophil surface marker expression of treated CD34+ HSPCs as assessed by FACS, after 14-day in culture with G-CSF (left column), Boskar3 (middle column), or Boskar4 (right column).

Supplementary Figure 26

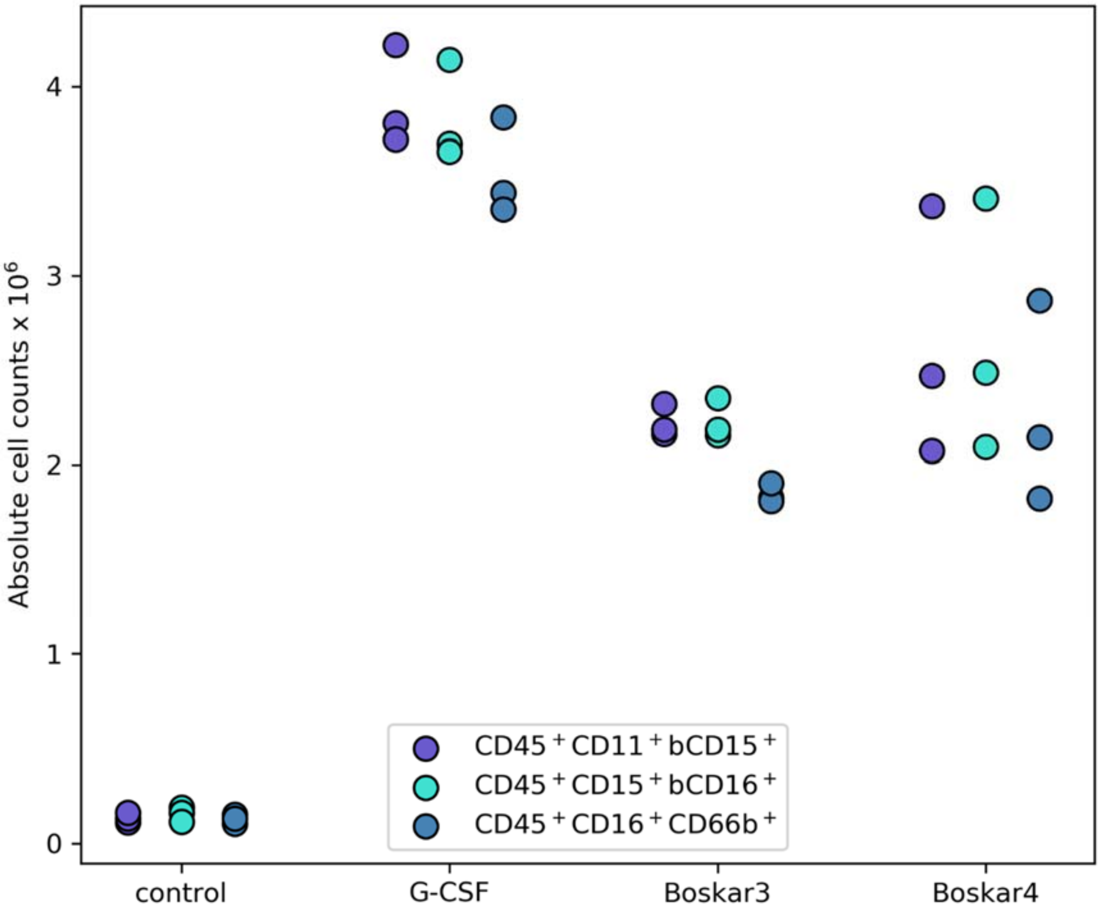

Supplementary Figure 26. Dot plot of cell counts with surface marker combinations in Fig. S25 for rhG-CSF, Boskar3, and Boskar4. Data show mean ± standard deviation across 3 biologically independent replicates.

Supplementary Figure 27

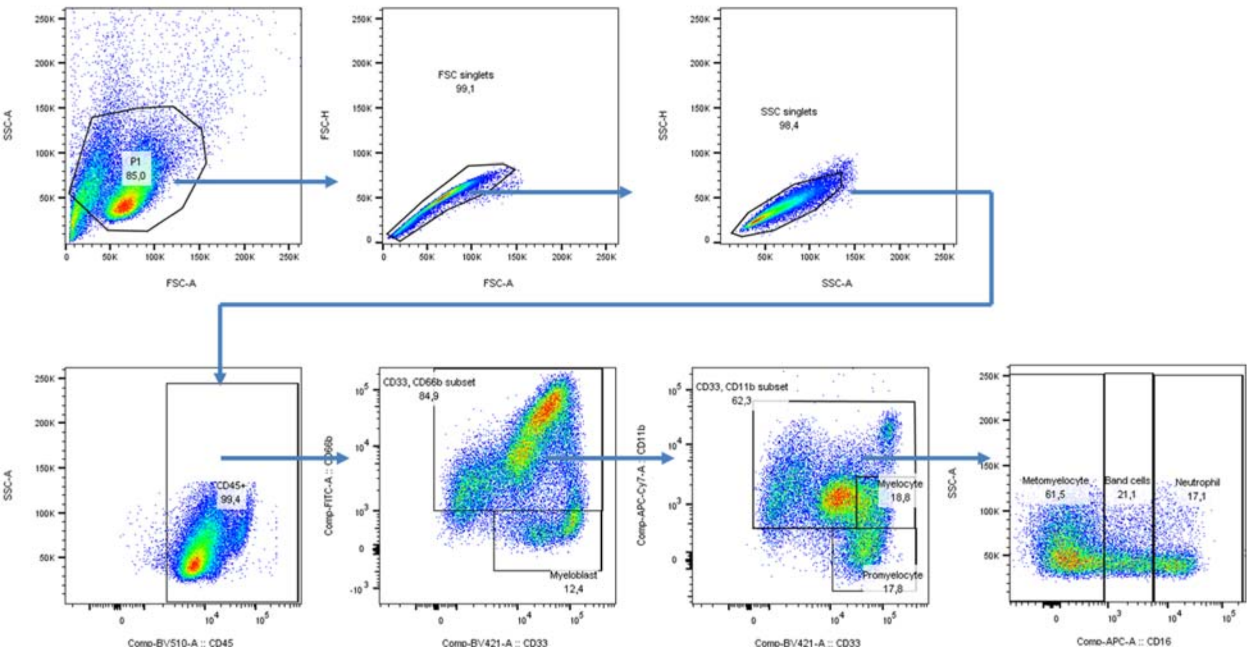

Supplementary Figure 27. Gating strategy of FACS analysis of differentiated HSPC cell in *in vitro* liquid differentiation culture.

Supplementary Figure 28

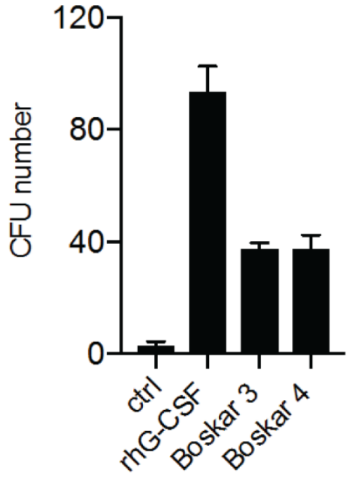

Supplementary Figure 28. Quantification of colony-forming units of HSPCs induced by either rhG-CSF, Boskar3, or Boskar4 in CD34$^+$ HSPCs after 14 days of culture in semi-solid medium (see Materials and Methods). Data show mean ± standard deviation.

Supplementary Figure 29

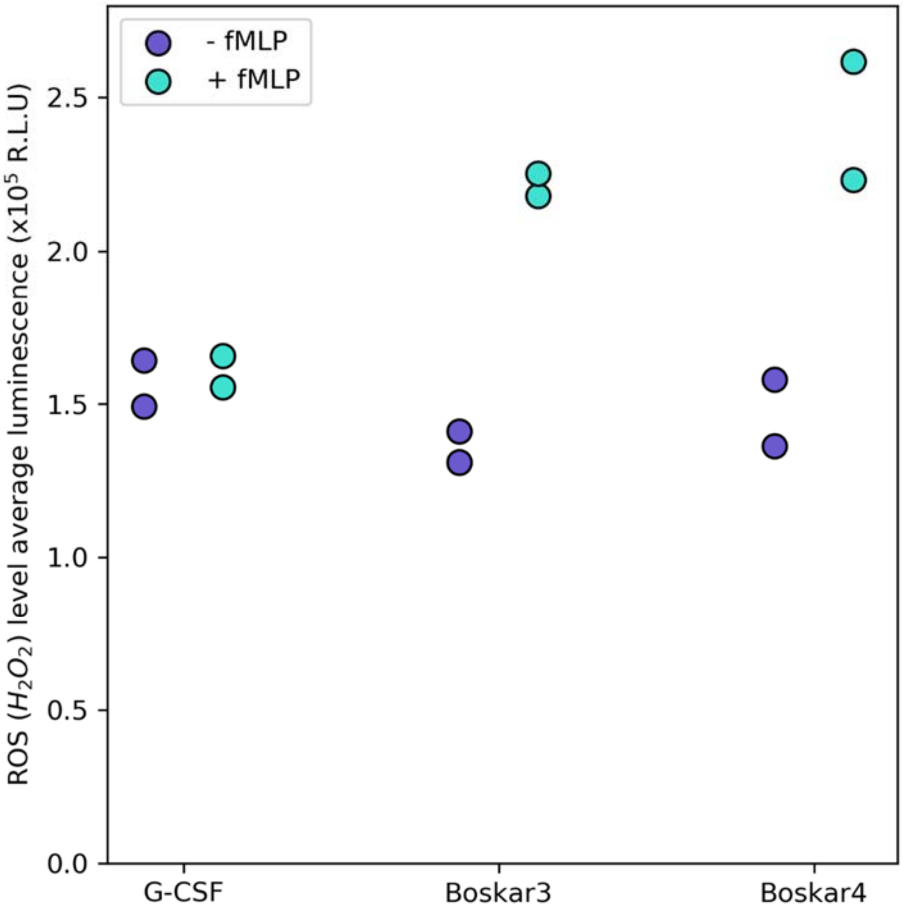

Supplementary Figure 29. Boskar3- and Boskar4-treated HSPCs generate reactive oxygen species. Reactive oxygen species (ROS) assay of granulocytes generated on day 14 of liquid culture treatment with either rhG-CSF, Boskar3, or Boskar4 (see Materials and Methods). Data show mean ± standard deviation of 3 biologically independent replicates.

Supplementary Figure 30

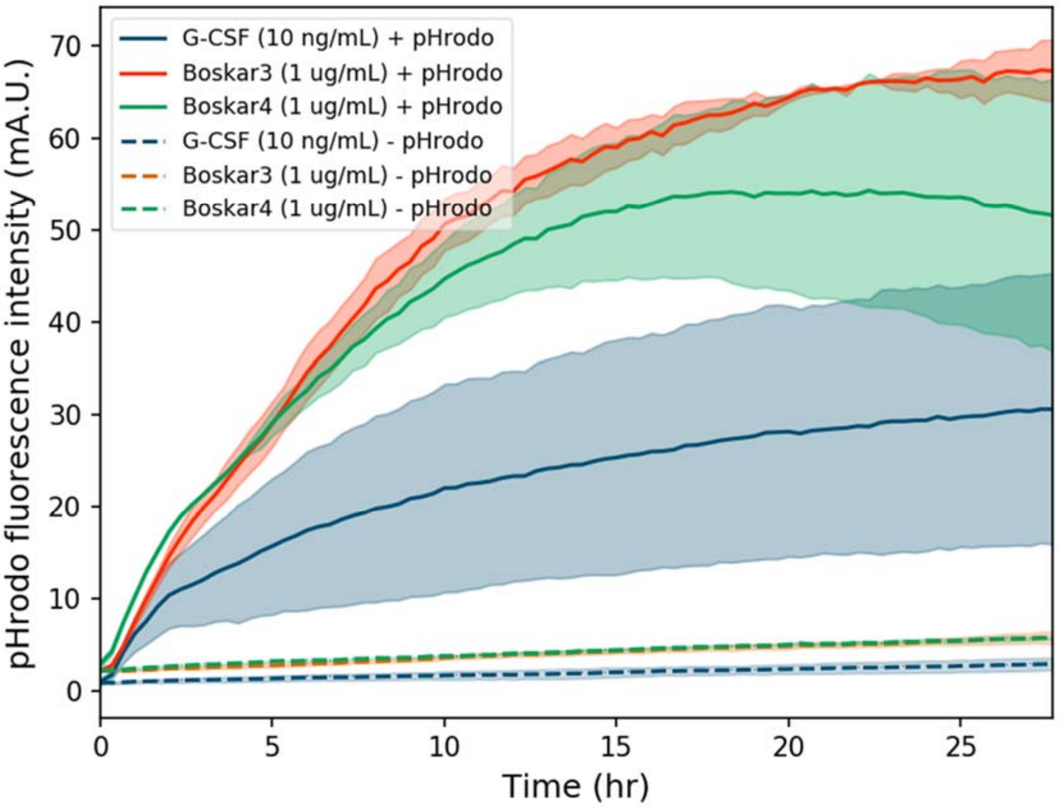

Supplementary Figure 30. Boskar3- and Boskar4-treated HSPCs possess phagocytic activity. Phagocytosis kinetic analysis of granulocytes generated on day 14 of liquid culture treatment with either rhG-CSF, Boskar3, Boskar4 (see Materials and Methods). Lines represent mean, shades represent ± standard deviation.

Supplementary Figure 31

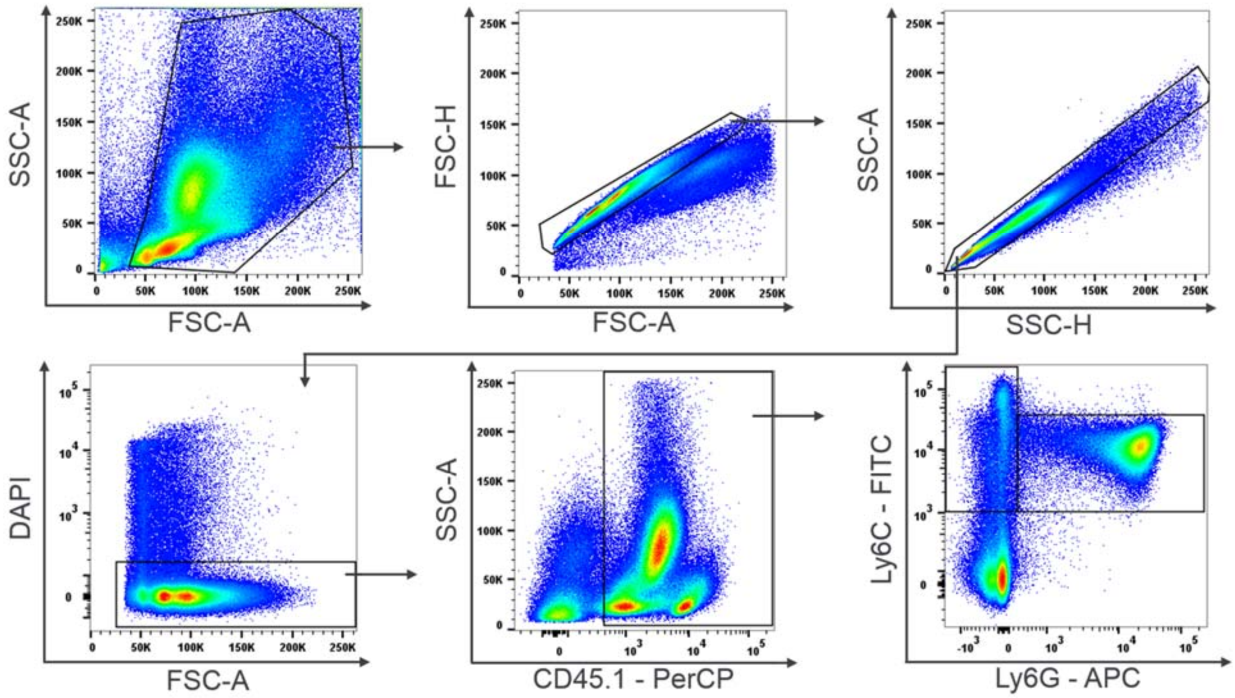

Supplementary Figure 31. Gating strategy for FACS analysis of neutrophils of treated mice. Cells were selected in FSC-A gated against SSC-A. Subsequently, singlet events were selected by gating of FSC-A versus FSC-H, followed by gating of SSC-H versus SSC-A. Then DAPI negative live cells were selected, gated against FSC-A. From these cells, CD45.1 positive cells were selected. Among these cells, $Ly6C^{+}Ly6G^{+}$ cells are neutrophils.

Supplementary Figure 32

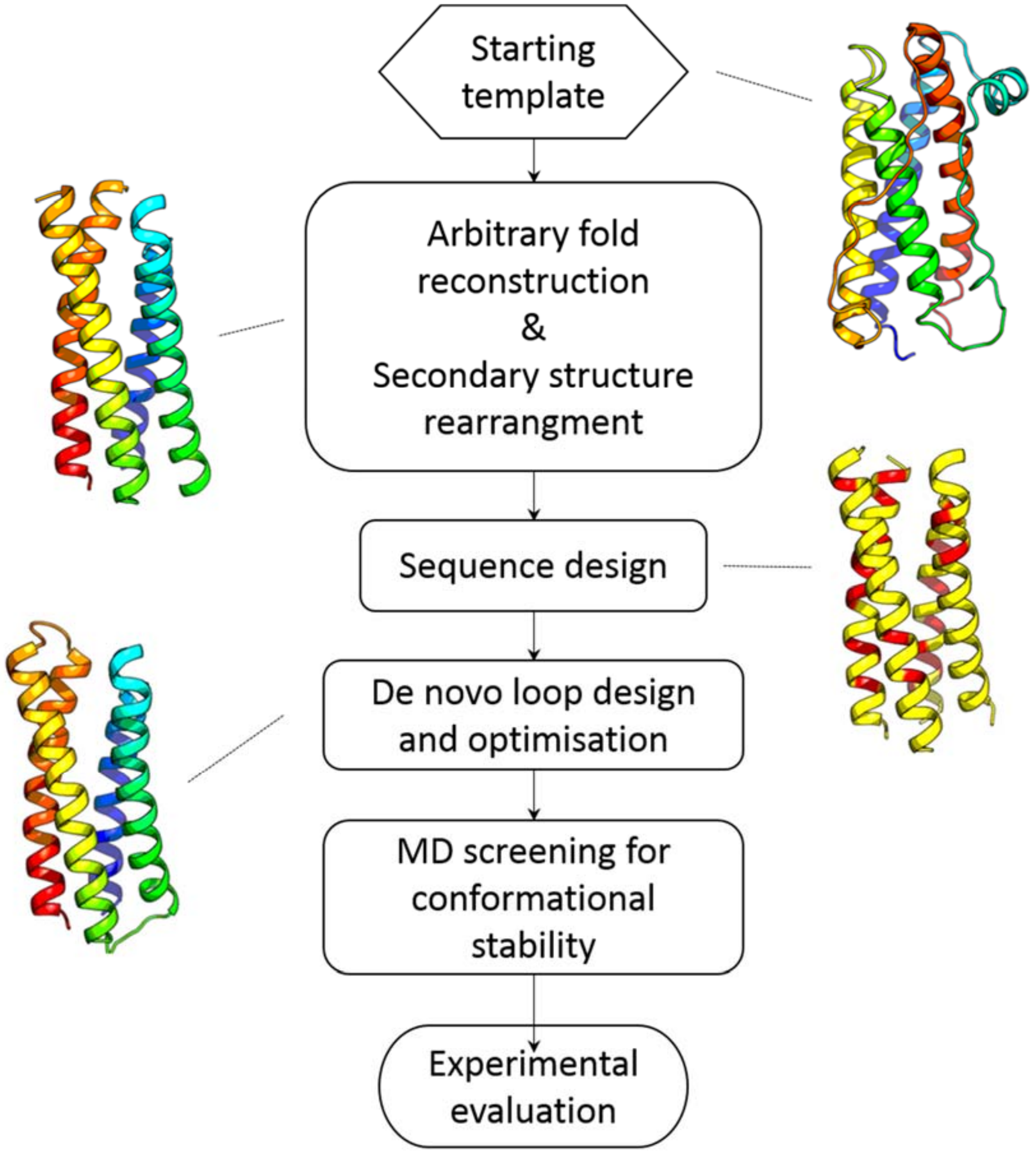

Supplementary Figure 32. A summary of the computational design workflow. G-CSF constituted the starting template. The main four-helix-bundle (4HB) was trimmed and the residues were renumbered to reorder the helices into an up-down 4HB. Residues at the core and the surface of the disjoint bundle were designed to improve the protein's stability. This was followed by construction of short loops *de novo*. MD simulations of these loops with restrained helices were used to rank the canidates, with conformational stability as the criterion to select optimal sequence at each splice site. Further MD simulations were run for global combinations of different loop compositions and bundle mutants, to rank the designs by structural stability. Finally, four designs were chosen for experimental evaluation of their structure and function.

Supplementary methods

Packing routine example in RosettaScripts:

```
<ROSETTASCRIPTS>
    <TASKOPERATIONS>
        <ReadResfile name=rrf filename=%%resfile%%/>
        <IncludeCurrent name=currentTask/>
        <RestrictToRepacking name=repackonly/>
        <InitializeFromCommandline name=cmdTask/>
    </TASKOPERATIONS>
    <FILTERS>
        <PackStat name=holes_1 threshold=%%pck_scr1%% chain=0 repeats=5/>
        <PackStat name=holes_2 threshold=%%pck_scr2%% chain=0 repeats=5/>
        <Rmsd name=rmsd threshold=5.0 confidence=1.0/>
        <ScoreType name=ttl_scr scorefxn=talaris2013 score_type=total_score
threshold=%%ttl_scr_thrshld%%/>
    </FILTERS>
    <MOVERS>
        <TaskAwareMinMover name=minmover_rpck scorefxn=soft_rep chi=1 bb=1
jump=1 task_operations=vectorTask,cmdTask,currentTask,repackonly/>
        <GreedyOptMutationMover name=grdy_opt_mut filter=ddG filter_delta=0.5
scorefxn=soft_rep relax_mover=minmover_rpck sample_type=low repack_shell=7.5
task_operations=rrf/>
        <Backrub name=backrub/>
        <BackrubDD name=backrubdd interface_distance_cutoff=8.0 moves=1000
sc_move_probability=0.1 scorefxn=talaris2013 small_move_probability=0.1
bbg_move_probability=0.1 task_operations=rrf/>
        <RepackMinimize name=des1 scorefxn_repack=soft_rep
scorefxn_minimize=soft_rep minimize_bb=0 task_operations=rrf/> minimize_rb=0
design_partner1=1 design_partner2=1 task_operations=rrf/>
        <RepackMinimize name=des2 scorefxn_repack=talaris2013
scorefxn_minimize=talaris2013 minimize_bb=0 task_operations=rrf/>
design_partner1=1 design_partner2=1 task_operations=rrf/>
        <RepackMinimize name=des3 minimize_bb=1 task_operations=rrf/>
design_partner1=1 design_partner2=1/>
        <FastRelax name=relax scorefxn=talaris2013 repeats=2
task_operations=currentTask,repackonly,cmdTask/>
        <ParsedProtocol name=design>
            <Add mover_name=des1/>
            <Add mover_name=backrub/>
            <Add mover_name=des1/>
            <Add mover_name=des2/>
            <Add mover_name=des2/>
            <Add mover_name=backrubdd/>
            <Add mover_name=des3/>
            <Add filter=holes_1/>
            <Add mover_name=relax/>
        </ParsedProtocol>
        <GenericMonteCarlo name=iterate filter_name=ttl_scr
scorefxn_name=talaris2013 mover_name=design trials=3/>
        <GenericMonteCarlo name=iterate_h filter_name=holes_1
scorefxn_name=talaris2013 mover_name=design trials=2/>
    </MOVERS>
    <PROTOCOLS>
        <Add mover=iterate/>
        <Add filter=holes_1/>
```

```
        <Add mover=iterate_h/>
        <Add filter=holes_2/>
        <Add filter=ttl_scr/>
    </PROTOCOLS>
</ROSETTASCRIPTS>

# ~/rstta_bin/rosetta_scripts.linuxgccrelease -database ~/rstta_db -s $fn -
nstruct 200 -out:prefix prefix_ -out:file:silent slnt_name -
out:file:scorefile score_name -parser:protocol core_redes.xml -
parser:script_vars resfile='csf_resfile' pck_scr1='0.55' pck_scr2='0.58'
ttl_scr_thrshld='-225.0' -mute all -ex1 -ex2; done; done

# csf_resfile:
NATRO
start

2 A ALLAAxc EX 1 EX 2 EX 3
4 A ALLAAxc EX 1 EX 2 EX 3
6 A PIKAA ATRK EX 1 EX 2 EX 3
8 A PIKAA LI EX 1 EX 2 EX 3
9 A PIKAA YRAHIML EX 1 EX 2 EX 3
13 A PIKAA YAQH EX 1 EX 2 EX 3
14 A POLAR EX 1 EX 2 EX 3
17 A PIKAA KA EX 1 EX 2 EX 3
20 A PIKAA KEQN EX 1 EX 2 EX 3
32 A PIKAA AS EX 1
21 A PIKAA ST EX 1
35 A PIKAA AT EX 1 EX 2 EX 3
37 A PIKAA QKRIV EX 1 EX 2 EX 3
40 A PIKAA MVIL EX 1 EX 2 EX 3
47 A PIKAA MIL EX 1 EX 2 EX 3
50 A APOLAR EX 1 EX 2 EX 3
73 A PIKAA ALVI EX 1 EX 2 EX 3
74 A PIKAA VLE EX 1 EX 2 EX 3
77 A PIKAA LVIM EX 1 EX 2 EX 3
82 A PIKAA A EX 1
84 A PIKAA A EX 1
92 A PIKAA SA EX 1
111 A PIKAA IVAQ EX 1 EX 2 EX 3
114 A PIKAA AV EX 1 EX 2
115 A PIKAA TVLYA  EX 1 EX 2 EX 3
116 A POLAR EX 1 EX 2 EX 3
117 A PIKAA VLIY EX 1 EX 2 EX 3
121 A PIKAA LMF EX 1 EX 2 EX 3
122 A PIKAA QHR EX 1 EX 2 EX 3
132 A PIKAA FLVIM EX 1 EX 2 EX 3
123 A PIKAA ARND EX 1 EX 2 EX 3
125 A PIKAA LM EX 1 EX 2 EX 3
127 A PIKAA QA EX 1 EX 2 EX 3
128 A PIKAA VA EX 1 EX 2
```

Example for alignment generation for Boskar4:

```
# loop generation
fout_n = "aln.ali"
fout_h = open(fout_n, 'w')
lp_len = 3
upstrm_seq = "LAGMLAEIHKGLYEYQARLQSLEGISPELGPALDALQLDVADFATTIWQQMEE"
dnstrm_seq = "LPQSFLLKALEQIRKIQGDGAALREKLAATYGGGQRAAAGVEIAAQLEAFLEKAYRILRH"
tmplt_name = "b4_seg_b"
trgt_bs_name = "b4_seg_b_"
tmplt_seq = """
>P1;%s
structureX:%s:FIRST:@:LAST:@:::-1.0:-1.0
LAGMLAEIHKGLYEYQARLQSLEGISPELGPALDALQLDVADFATTIWQQMEE---
LPQSFLLKALEQIRKIQGDGAALREKLAATYGGGQRAAAGVEIAAQLEAFLEKAYRILRH*""" %
(tmplt_name, tmplt_name)
res_lst_9 = ["G", "D", "P", "S", "L", "N", "T", "E", "K"]
res_lst_8 = ["G", "D", "P", "S", "L", "N", "T", "K"]
tri_lp_lst = [x+y+z for x in res_lst_9 for y in res_lst_9 for z      in
res_lst_9]
tetra_lp_lst = [w+x+y+z for w in res_lst_8 for x in res_lst_8 for y in
res_lst_8 for z in res_lst_8]
if lp_len == 3:
        for lp_idx in xrange(len(tri_lp_lst)):
                tmp_str = ">P1;%s_%.3d\nsequence::::::::\n" % (trgt_bs_name,
lp_idx)
                tmp_str += upstrm_seq + tri_lp_lst[lp_idx] + dnstrm_seq+'*\n'
                fout_h.write(tmp_str)
elif lp_len == 4:
        for lp_idx in xrange(len(tetra_lp_lst)):
                tmp_str = ">P1;%s_%.4d\nsequence::::::::\n" % (trgt_bs_name,
lp_idx)
                tmp_str += upstrm_seq + tetra_lp_lst[lp_idx] +
dnstrm_seq+'*\n'
                fout_h.write(tmp_str)
fout_h.write(tmplt_seq)
fout_h.flush()
fout_h.close()
```

Example alignment input:

```
# example modeller input of 9**3 sequence combinations for a 3-residue loop:
>P1;boskar_000
sequence:::::::::
LAGMLAEIHKGLYEYQARLQSLEGISPELGPALDALQLDVADFATTIWQQMEEGGGLPQSFLLKALEQIRKIQGDGA
ALREKLAATYGGGQRAAAGVEIAAQLEAFLEKAYRILRHLA*
>P1;boskar_001
sequence:::::::::
LAGMLAEIHKGLYEYQARLQSLEGISPELGPALDALQLDVADFATTIWQQMEEGGDLPQSFLLKALEQIRKIQGDGA
ALREKLAATYGGGQRAAAGVEIAAQLEAFLEKAYRILRHLA*
.
.
.
>P1;boskar_727
sequence:::::::::
LAGMLAEIHKGLYEYQARLQSLEGISPELGPALDALQLDVADFATTIWQQMEEKKELPQSFLLKALEQIRKIQGDGA
ALREKLAATYGGGQRAAAGVEIAAQLEAFLEKAYRILRHLA*
>P1;boskar_728
sequence:::::::::
LAGMLAEIHKGLYEYQARLQSLEGISPELGPALDALQLDVADFATTIWQQMEEKKKLPQSFLLKALEQIRKIQGDGA
ALREKLAATYGGGQRAAAGVEIAAQLEAFLEKAYRILRHLA*
>P1;core_rnd02_out
structureX:core_rnd02_out:FIRST:@:LAST:@:::-1.0:-1.0
*
```

Example of loop building in Modeller:

```
# loop model building
from modeller import *
from modeller.automodel import *
from modeller.parallel import *
env = environ()
env.io.atom_files_directory = ['.', '../atom_files']
for i in xrange(1, 2000):
    a = automodel(env,
                  alnfile  = 'aln.ali',
                  knowns   = ('tmplt'),
                  sequence = 'boskar_a4_b3_%.4d' % i)
    a.auto_align()
    a.library_schedule = autosched.slow
    a.max_var_iterations = 300
    a.md_level = refine.slow
    a.repeat_optimization = 3
    a.max_molpdf = 1e6
    a.make()
```
