# Supplementary material for: A topological refactoring design strategy yields highly stable granulopoietic proteins
Source: Nat Commun. 2022 May 26;13:2948. doi: 10.1038/s41467-022-30157-2 (PMC9135769; doi:10.1038/s41467-022-30157-2)
Supplement: Supplementary file 3 — Description of Additional Supplementary Files [file 41467_2022_30157_MOESM3_ESM.pdf]

Description of Additional Supplementary Files

File Name: Supplementary Movie 1
Description: Representative time-lapse analysis of NFS-60 cells cultured without treatment (PBS only) for 136 h.

File Name: Supplementary Movie 2
Description: Representative time-lapse analysis of NFS-60 cells cultured with rhGCSF (10 ng/mL) treatment for 136 h.

File Name: Supplementary Movie 3
Description: Representative time-lapse analysis of NFS-60 cells cultured with Boskar3 (4 µg/mL) treatment for 136 h.

File Name: Supplementary Movie 4
Description: Representative time-lapse analysis of NFS-60 cells cultured with Boskar4 (4 µg/mL) treatment for 136 h.
