# Supplementary material for: A topological refactoring design strategy yields highly stable granulopoietic proteins
Source: Nat Commun. 2022 May 26;13:2948. doi: 10.1038/s41467-022-30157-2 (PMC9135769; doi:10.1038/s41467-022-30157-2)
Supplement: Supplementary file 8 — Reporting Summary [file 41467_2022_30157_MOESM8_ESM.pdf]

nature research

Corresponding author(s): Julia Skokowa, Mohammad ElGamacy

Last updated by author(s): Apr 18, 2022

Reporting Summary

Nature Research wishes to improve the reproducibility of the work that we publish. This form provides structure for consistency and transparency in reporting. For further information on Nature Research policies, see our Editorial Policies and the Editorial Policy Checklist.

Statistics

For all statistical analyses, confirm that the following items are present in the figure legend, table legend, main text, or Methods section.

n/a	Confirmed	
☐	☒	The exact sample size (*n*) for each experimental group/condition, given as a discrete number and unit of measurement
☐	☒	A statement on whether measurements were taken from distinct samples or whether the same sample was measured repeatedly
☐	☒	The statistical test(s) used AND whether they are one- or two-sided<br>*Only common tests should be described solely by name; describe more complex techniques in the Methods section.*
☐	☒	A description of all covariates tested
☒	☐	A description of any assumptions or corrections, such as tests of normality and adjustment for multiple comparisons
☐	☒	A full description of the statistical parameters including central tendency (e.g. means) or other basic estimates (e.g. regression coefficient) AND variation (e.g. standard deviation) or associated estimates of uncertainty (e.g. confidence intervals)
☐	☒	For null hypothesis testing, the test statistic (e.g. *F*, *t*, *r*) with confidence intervals, effect sizes, degrees of freedom and *P* value noted<br>*Give P values as exact values whenever suitable.*
☒	☐	For Bayesian analysis, information on the choice of priors and Markov chain Monte Carlo settings
☒	☐	For hierarchical and complex designs, identification of the appropriate level for tests and full reporting of outcomes
☒	☐	Estimates of effect sizes (e.g. Cohen's *d*, Pearson's *r*), indicating how they were calculated

Our web collection on statistics for biologists contains articles on many of the points above.

Software and code

Policy information about availability of computer code

Data collection	Modeller, RosettaDesign, OpenMM, NAMD, Bruker topspin, TALOS, Jasco spectral analysis sofware, scikit-image, NumPy.
Data analysis	Bruker topspin, Sparky, CCPN, TALOS, Biacore X100 evaluation software, SciPy, VMD, TIDE webtool, FACSDiva, Systat, IncuCyte S3 software.

For manuscripts utilizing custom algorithms or software that are central to the research but not yet described in published literature, software must be made available to editors and reviewers. We strongly encourage code deposition in a community repository (e.g. GitHub). See the Nature Research guidelines for submitting code & software for further information.

Data

Policy information about availability of data

All manuscripts must include a data availability statement. This statement should provide the following information, where applicable:

- Accession codes, unique identifiers, or web links for publicly available datasets
- A list of figures that have associated raw data
- A description of any restrictions on data availability

NMR structure deposited on the PDB; all protein sequences are described, all of the raw experimental results are available upon request.

Field-specific reporting

Life sciences study design

All studies must disclose on these points even when the disclosure is negative.

Sample size	*Describe how sample size was determined, detailing any statistical methods used to predetermine sample size OR if no sample-size calculation was performed, describe how sample sizes were chosen and provide a rationale for why these sample sizes are sufficient.*
Data exclusions	*Describe any data exclusions. If no data were excluded from the analyses, state so OR if data were excluded, describe the exclusions and the rationale behind them, indicating whether exclusion criteria were pre-established.*
Replication	*Describe the measures taken to verify the reproducibility of the experimental findings. If all attempts at replication were successful, confirm this OR if there are any findings that were not replicated or cannot be reproduced, note this and describe why.*
Randomization	*Describe how samples/organisms/participants were allocated into experimental groups. If allocation was not random, describe how covariates were controlled OR if this is not relevant to your study, explain why.*
Blinding	*Describe whether the investigators were blinded to group allocation during data collection and/or analysis. If blinding was not possible, describe why OR explain why blinding was not relevant to your study.*

Reporting for specific materials, systems and methods

We require information from authors about some types of materials, experimental systems and methods used in many studies. Here, indicate whether each material, system or method listed is relevant to your study. If you are not sure if a list item applies to your research, read the appropriate section before selecting a response.

Materials & experimental systems

n/a	Involved in the study	
☐	☒	Antibodies
☐	☒	Eukaryotic cell lines
☒	☐	Palaeontology and archaeology
☐	☒	Animals and other organisms
☐	☒	Human research participants
☒	☐	Clinical data
☒	☐	Dual use research of concern

Methods

n/a	Involved in the study	
☒	☐	ChIP-seq
☐	☒	Flow cytometry
☒	☐	MRI-based neuroimaging

Antibodies

Antibodies used

Biolegend #303416; Mouse anti-human CD45 (clone HI30) BV510, Biolegend #304036; Mouse anti-human CD11b APC-Cy7 (clone ICRF44), BD #557754; Mouse anti-human CD15 PE (clone W6D3), Biolegend #323006; Mouse anti-human CD16 APC (clone 3G8), BD #561248; Mouse anti-human CD66b FITC (clone G10F5), Biolegend #305104

Validation

Mouse anti-human CD45 BV510 was validated using human peripheral blood lymphocytes. REF: Oeztuerk-Winder F, et al. 2012. EMBO J. 31:3431. (Flow Cytometry);

Mouse anti-human CD11b APC-Cy7 was validated using human whole blood with lyzed erythrocytes. REF: Hogg N, Horton MA. Myeloid antigens: New and previously defined clusters. In: McMichael AJ. A.J. McMichael .. et al., ed. Leucocyte typing III : white cell differentiation antigens. Oxford New York: Oxford University Press; 1987:576-602.;

Mouse anti-human CD15 PE was validated on human peripheral blood granulocytes. REF: Raffray L, et al. 2016. PLoS One. 11:e0165716.;

Mouse anti-human CD16 APC was validated using Rhesus macaque lysed whole blood. REF: Stroncek DF, Skubitz KM, Plachta LB, et al. Alloimmune neonatal neutropenia due to an antibody to the neutrophil Fc-gamma receptor III with maternal deficiency of CD16 antigen. Blood. 1991; 77(7):1572-1580.;

Mouse anti-human CD66b was validated using human peripheral whole blood granulocytes. REF: Ackermann M, et al. 2018. Nat Commun. 9:5088.

Eukaryotic cell lines

Policy information about cell lines

Cell line source(s)	NFS-60 cell line
Authentication	NFS-60 cell line was purchased by ATCC, NFS-60 (ATCC® CRL-1838)

Mycoplasma contamination	NFS-60 cell line was tested negative for mycoplasma contamination
Commonly misidentified lines (See ICLAC register)	*Name any commonly misidentified cell lines used in the study and provide a rationale for their use.*

Animals and other organisms

Policy information about studies involving animals; ARRIVE guidelines recommended for reporting animal research

Laboratory animals	C57BL/6 mice
Wild animals	*Provide details on animals observed in or captured in the field; report species, sex and age where possible. Describe how animals were caught and transported and what happened to captive animals after the study (if killed, explain why and describe method; if released, say where and when) OR state that the study did not involve wild animals.*
Field-collected samples	*For laboratory work with field-collected samples, describe all relevant parameters such as housing, maintenance, temperature, photoperiod and end-of-experiment protocol OR state that the study did not involve samples collected from the field.*
Ethics oversight	German federal and state 686 regulations (Regierungspräsidium Tübingen, K3/17)

Note that full information on the approval of the study protocol must also be provided in the manuscript.

Human research participants

Policy information about studies involving human research participants

Population characteristics	healthy donors
Recruitment	Informed written consent was obtained from all participants of this study.
Ethics oversight	Study approval was obtained from the Ethical Review Board of the Medical Faculty, University of Tübingen.

Note that full information on the approval of the study protocol must also be provided in the manuscript.

Flow Cytometry

Plots

Confirm that:

- [x] The axis labels state the marker and fluorochrome used (e.g. CD4-FITC).
- [x] The axis scales are clearly visible. Include numbers along axes only for bottom left plot of group (a 'group' is an analysis of identical markers).
- [x] All plots are contour plots with outliers or pseudocolor plots.
- [x] A numerical value for number of cells or percentage (with statistics) is provided.

Methodology

Sample preparation	CD34+ hematopoietic stem/progenitor cells (HSPCs) were isolated by MACS separation (Miltenyi) from bone marrow mononuclear cell fraction. Isolated CD34+ HSPCs were expanded in vitro for aprx. 4 days before performing in vitro granulocytic differentiation for 14 days. Cell staining for flow cytometry was conducted for 30 min on ice, washed twice with PBS supplemented with 2% BSA and stored at 4 °C before measurement.
Instrument	BD FACS Canto II
Software	FACS Diva II and Flowjo V10
Cell population abundance	cell population abundance was high enough to be able to estimate it correctly by FACS
Gating strategy	The gating was set according to unstained and FMO samples.<br>All indicated populations were gated from Singlets and CD45+ fraction.<br>Neutrophils are defined as:<br>CD45+CD11b+CD15+<br>CD45+CD15+CD16+<br>CD45+CD16+CD66b+

- [x] Tick this box to confirm that a figure exemplifying the gating strategy is provided in the Supplementary Information.
